# Dual‐Tasking Exacerbates Force and Neural Control Unsteadiness in Older Adults With Sarcopenia

**DOI:** 10.1002/jcsm.70397

**Published:** 2026-10-05

**Authors:** Lucas B. R. Orssatto, Brian C. Clark, David Scott, Hélio V. Cabral, Gabriel L. Fernandes, Robin M. Daly

**Affiliations:** ^1^ Centre for Sensorimotor Performance, School of Human Movement and Nutrition Sciences The University of Queensland Brisbane Queensland Australia; ^2^ Institute for Physical Activity and Nutrition, School of Exercise and Nutrition Sciences Deakin University Burwood Victoria Australia; ^3^ Ohio Musculoskeletal and Neurological Institute (OMNI) Ohio University Athens Ohio USA; ^4^ Department of Biomedical Sciences, Heritage College of Osteopathic Medicine Ohio University Athens Ohio USA; ^5^ Department of Medicine, School of Clinical Sciences at Monash Health Monash University Clayton Victoria Australia; ^6^ School of Physical Education and Sports Universidade Federal do Rio de Janeiro Rio de Janeiro Brazil; ^7^ Biomedical Engineering Program (COPPE) Universidade Federal do Rio de Janeiro Rio de Janeiro Brazil; ^8^ Postgraduate Program in Rehabilitation Sciences Universidade Federal do Rio de Janeiro Rio de Janeiro Brazil; ^9^ Physical Activity, Sport and Exercise Research (PASER) Theme, Faculty of Health Southern Cross University Gold Coast Queensland Australia

**Keywords:** aging, common synaptic input, high‐density electromyography, masters athletes, motor unit

## Abstract

**Background:**

Sarcopenia is associated with impaired physical function. Dual‐task conditions, which increase cognitive demand during motor performance, may reveal deficits in neuromuscular control that are not evident during isolated motor tasks. We investigated whether older adults with sarcopenia exhibit poorer force and neural control (i.e., greater force and motor unit inter‐spike variability and altered common synaptic input) during single‐ and dual‐task conditions compared with non‐sarcopenic controls and masters athletes.

**Methods:**

Sarcopenia was defined using Sarcopenia Definitions and Outcomes Consortium criteria (low grip strength and slow gait). Participants (11 sarcopenic, 22 controls and 19 masters athletes; 74.3 ± 7.3 years; 50% female) performed sustained isometric ankle dorsiflexion contractions at 30% of maximal voluntary torque, without (single‐task) and with concurrent serial number subtraction (dual‐task). Motor unit spike trains were decomposed from high‐density surface electromyograms. Outcomes included torque and inter‐spike interval coefficient of variation (index of discharge regularity linked to torque steadiness) and intramuscular coherence: delta (1–5 Hz; low‐frequency common input related to force fluctuations), alpha (5–15 Hz; which may include tremor‐related, spinal and afferent contributions) and beta (15–35 Hz; commonly associated with corticospinal input) bands.

**Results:**

Individuals with sarcopenia had higher torque coefficient of variation (worse steadiness) than controls in single‐ and dual‐task by 45% and 84% and athletes by 39% and 105%, respectively. In individuals with sarcopenia, coefficient of variation rose ~35% from single‐ to dual‐task. Mean discharge rate (a proxy of neural drive) increased ~2.6% during dual‐tasking in all groups, without between‐group differences. Inter‐spike interval coefficient of variation increased by 5.5% in sarcopenic, was unchanged in controls and decreased 4.1% in athletes. Delta‐band coherence decreased 5.5% across all groups, whereas alpha‐band coherence increased in sarcopenic only (20.6%). Beta‐band coherence increased 14.5% in sarcopenic but decreased 8.4% in controls and 13.3% in athletes.

**Conclusions:**

Older adults with sarcopenia exhibit poorer force and neural control steadiness, and both deficits worsen under cognitive load. These changes are accompanied by alterations in common synaptic input, particularly increases in alpha and beta bands in individuals with sarcopenia, likely contributing to their greater force unsteadiness during dual‐tasking. Neural control unsteadiness during dual‐task performance may therefore represent a neural feature of sarcopenia‐related functional impairment. Assessing neuromuscular control during cognitively demanding tasks may improve detection of neural dysfunction and identify mechanistic targets for interventions aimed at preserving mobility in older adults. These findings support expanding muscle‐centric views of sarcopenia to include neural mechanisms of motor control.

## 1 Introduction

Sarcopenia is a progressive skeletal muscle disease that has been strongly linked to frailty, falls, disability and loss of independence in older adults [1]. Although it is commonly defined by low muscle mass, low muscle strength and impaired physical function [1, 2, 3], declines in force‐producing capacity and control are unlikely to be explained by muscle morphology alone [4, 5, 6, 7]. Deterioration in the neuromotor system [4, 5, 8, 9, 10] is functionally relevant because stable force production depends on the nervous system's ability to generate and maintain coordinated neural drive to muscle [11]. Consistent with this, older adults exhibit greater force fluctuations and more variable motor unit discharge during submaximal sustained muscle contraction than young adults, suggesting that impaired neural control is a key feature of age‐related reductions in motor performance [7, 8, 12, 13, 14, 15, 16]. These deficits may be especially important under conditions that more closely reflect everyday function or activities of daily living.

Many real‐world activities relevant to falls and independence, such as walking, rising from a chair or carrying objects, are performed, while attention is divided between motor and cognitive demands [12, 13]. Under such dual‐task conditions, older adults commonly move more slowly, show greater instability and are more likely to make contact with obstacles [17, 18, 19, 20], with impaired dual‐task performance also linked to both prior falls and future fall risk [17, 21]. Experimental work further shows that adding a cognitive task during steady contractions increases the variability in force and motor unit discharge rates, indicating that cognitive load can degrade the neural control of force [12, 13]. Older adults with sarcopenia also demonstrate poorer dual‐task gait performance than those without sarcopenia [20]. In contrast, older adults engaged in long‐term sports training (i.e., masters athletes), a context that typically demands multitasking, exhibit better resilience to dual‐task interference [22, 23]. We have previously shown that intrinsic motoneuron excitability differs across these phenotypes (i.e., sarcopenic, non‐sarcopenic controls and masters athletes) and contributes to poor physical function [24]. However, whether these phenotypic differences in physical function are also accompanied by altered neural drive and force control, particularly under dual‐task conditions, is not known.

High‐density surface electromyography (HD‐EMG) combined with motor unit decomposition enables detailed examination of motor unit discharge behaviour during voluntary contractions [25]. By integrating measures of torque steadiness and motor unit discharge properties, it is possible to quantify both the steadiness of motor output and the organisation of common synaptic inputs to the motoneuron pool [11, 12]. Common synaptic input refers to neural signals that are sent to many motor units, particularly from descending corticospinal pathways [26, 27], such that their resultant activity is highly correlated [28, 29]. This shared input contributes substantially to the effective neural command to the muscle, enabling smooth and coordinated force production [11, 30]. Surface EMG amplitude reflects only the overall amount of electrical activity a muscle produces; it does not show how that activity is organised^S41^. Motor unit decomposition identifies when each motor unit discharges, and therefore how closely different motor units are driven together [11, 30, 31, 32]. Because it is this shared input, rather than the total amount of muscle activity, that is converted into steady force, a reduction in shared input produces larger fluctuations in motor output and poorer force control [11]. Coherence between motor units is examined separately in three frequency bands, each reflecting a different source of common input. Delta (1–5 Hz) represents common drive related to force fluctuations, alpha (5–15 Hz) represents subcortical and spinal contributions linked to physiological tremor and beta (15–35 Hz) represents corticospinal coupling [11, 30, 33]. Examining the bands separately is informative because each reflects input from a different level of the nervous system. Comparing across bands therefore indicates not only whether the organisation of neural drive is altered in sarcopenia or under cognitive load but where in the nervous system those changes may arise.

The aim of this study was to characterise neuromuscular control during submaximal isometric contractions performed under single‐ and dual‐task conditions in older adults with sarcopenia, non‐sarcopenic controls and masters athletes. We hypothesised that participants with sarcopenia would exhibit poorer torque steadiness and greater motor unit discharge variability, particularly under cognitive load, whereas masters athletes would demonstrate the most stable performance. We further hypothesised that dual‐tasking would alter common synaptic input in individuals with sarcopenia, consistent with a reduced capacity to maintain coordinated neural drive when cognitive demands are increased. These physiological differences were anticipated given the motoneuron deterioration/remodelling and altered afferent input that may accompany sarcopenia [4, 5, 24], and the adaptable neural control fostered by the masters athletes' varied training.

## 2 Methods

### 2.1 Participants

This cross‐sectional study was conducted in the same cohort described previously [24] but used a distinct dual‐task isometric protocol and motor unit analysis to address a separate mechanistic question. Full participant characterisation is reported elsewhere [24]. Eligibility criteria included age ≥ 65 years, no use of medications affecting noradrenergic or serotonergic systems (e.g., psychiatric medications), no sex hormone replacement therapy or performance‐enhancing drugs and no lower‐limb musculoskeletal or neurological conditions that could affect testing outcomes. Individuals with sarcopenia and controls required a body mass index ≤ 30 kg/m² [2]. Masters athletes had ≥ 10 years of competitive sport participation (with < 1 year interruption) and engagement in competition within the previous year. Controls were non‐sarcopenic and reported ≤ 1 structured exercise session per week in the previous year. The study was approved by the Deakin University Human Research Ethics Committee (2023–241) and conducted in accordance with the Declaration of Helsinki. All participants provided written informed consent.

### 2.2 Sarcopenia Screening

Participants underwent a multistage screening protocol to determine sarcopenia status [24]. Initial screening used the five‐item SARC‐F questionnaire^S42^. Individuals scoring ≥ 2 completed additional questions on walking ability and load carrying, and a video‐based Five Times Sit‐to‐Stand (5STS) test. Those reporting slow walking, difficulty lifting small loads and a 5STS time > 15 s were invited for in‐person assessment. All participants underwent the laboratory screening workflow, allowing the sarcopenia group to be classified and confirming that all participants in the control and masters groups were non‐sarcopenic. Sarcopenia was diagnosed according to Sarcopenia Definitions and Outcomes Consortium (SDOC) criteria [2], defined by low muscle strength (handgrip strength < 20 kg for females or < 35.5 kg for males) and impaired physical performance (usual gait speed < 0.8 m · s^−1^). Handgrip strength was measured with a digital dynamometer (Jamar Plus, United States) with participants seated, shoulder neutral and elbow flexed at 90°. Participants performed two maximal contractions (3–5 s) with the dominant hand, and the highest value was used.

### 2.3 Neuromuscular Assessments

Participants were seated upright with their preferred leg secured to an ankle dynamometer (DinamometroGC, OT Bioelettronica, Italy) with the hip at ~70° flexion, the knee at 0° extension and the ankle at 5° plantarflexion (0° anatomical position). The foot was secured with a strap placed across the proximal phalanges, and the knee was secured to prevent movement. The skin over the tibialis anterior was shaved, abraded (Everi preparation paste, Spes Medica, Italy) and cleaned with 70% isopropyl alcohol. Two HD‐EMG 64‐channel electrode grids (8‐mm interelectrode distance; HD08MM1106, OT Bioelettronica) were placed over the centre of the muscle belly of the tibialis anterior, covering its superior and inferior regions (muscle length was measured and an approximately similar distance was allowed for top and bottom extremities), aligned with the fibre direction, using bi‐adhesive foam and conductive paste (AC cream, Spes Medica). A compression bandage secured electrode contact and a dampened strap electrode (WS1.1, OT Bioelettronica) placed around the ankle served as reference. Real‐time visual torque feedback was displayed on a monitor ~1 m from the participants' eyes (screen size 15.6″, *y*‐axis scaling 0%–35% relative to peak torque), with gain and settings identical across participants and conditions. The tibialis anterior was selected because it is the primary dorsiflexor responsible for foot clearance during gait (a component of current operational definitions of sarcopenia [2, 3, 34, 35], and one whose impaired control is linked to falls) and because it is better suited to motor unit decomposition than other lower‐limb muscles in older adults [36]. A more detailed rationale has been reported previously [24].

Data collection began 10–15 min after the preceding session, which comprised the ramped‐contraction protocol reported previously [three 20‐s triangular contractions at 20%, 40% and 60% of maximal voluntary torque (MVT), with rest periods of 90, 120 and 150 s, respectively] [24]. For familiarisation, participants performed shorter trapezoidal contractions (3‐s ramp up, 10‐s plateau, 3‐s ramp down) at 30% MVT [24]. They were also familiarised with the cognitive task, in which they were asked to subtract 7, continuously, starting from a number between 70 and 99 (excluding multiples of 7). All participants were able to perform the subtraction task taking less than 3 s to provide an answer after each subtraction. After 5 min rest, participants performed six contractions at 30% MVT (3‐s ramp up, 20‐s plateau, 3‐s ramp down; 2‐min rest), three under single‐task (motor task alone) and three under dual‐task (motor + cognitive task) conditions in alternating randomised order (Figure 1). Trials were considered invalid if subtraction was interrupted for > 3 s; errors of ± 1 were accepted, whereas more than one error of ± 2 invalidated the trial. One additional trial per condition was permitted only when a trial was invalidated (subtraction interrupted > 3 s or contraction interrupted at any time), ensuring up to three valid trials without inducing fatigue. Single‐ and dual‐task trials were alternated in randomised order to ensure that any residual fatigue would be distributed evenly across conditions. During these contractions, HD‐EMG was recorded in monopolar mode, amplified (205×), band‐pass filtered (10–500 Hz) and digitised at 2000 Hz using a 16‐bit wireless amplifier (Muovi+, OT Bioelettronica) and the OTBioLab+ software (v1.5.9.2) with real‐time visual torque feedback.

**FIGURE 1:**
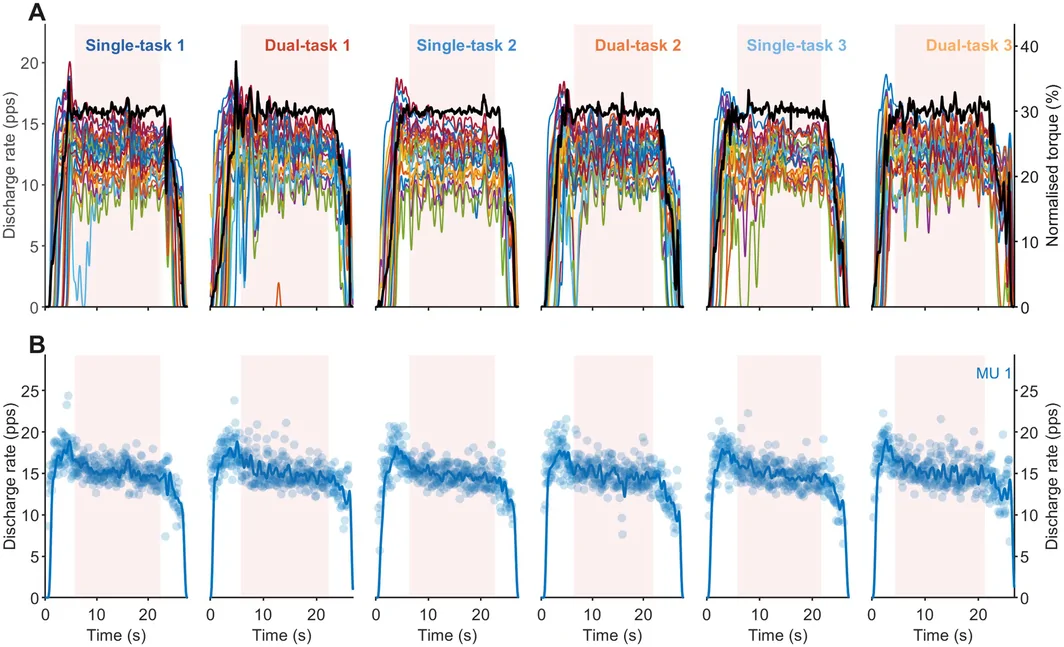
Study design and individual sarcopenic participant data. (Panel A) Smoothed discharge rates of individual motor units (coloured lines) identified from the tibialis anterior during isometric ankle dorsiflexion at 30% of maximal voluntary torque. Each panel corresponds to one trapezoidal contraction, with single‐task and dual‐task conditions displayed separately. The black line represents the torque trace (right *y*‐axis), expressed as a percentage of maximal voluntary torque. The shaded red region denotes the analysis window selected at the torque plateau. (Panel B) Instantaneous discharge rate (dots) and smoothed discharge rate (solid line) of the earliest‐recruited motor unit across the six contractions. The shaded red region indicates the analysis window used for coherence estimation. Units that did not exhibit continuous firing throughout the analysis window (e.g., light blue unit from contraction 1, orange unit from contraction 2 and green unit from contraction 5) were excluded from statistical analyses but are still displayed in Panel A for transparency and to illustrate the full pool of identified motor units.

### 2.4 Torque and HD‐EMG Analyses

The load cell signal from the ankle dynamometer was digitised, amplified (205×) and low‐pass filtered offline at 15 Hz. HD‐EMG signals were processed offline using the DEMUSE software^S43^ and band‐pass filtered (20–500 Hz) with a second‐order zero‐lag Butterworth filter. Torque was estimated using the equation provided in the manufacturer's manual and available with the open‐source statistical code (DinamometroGC, OT Bioelettronica, Italy). Motor unit spike trains were identified using the convolutive kernel compensation algorithm^S43^. Separation vectors were then applied to concatenated HD‐EMG signals to track motor units across trials and conditions^S44^. Duplicate units (> 30% common discharge times) were removed^S45^. An experienced investigator (G. L. F.), blinded to group and condition, manually corrected missed or misidentified spikes. Only motor units with a pulse‐to‐noise ratio ≥ 30 dB were included. Units with noncontinuous firing during the analysis window were not included in the analyses.

Instantaneous discharge rates were calculated as the inverse of inter‐spike intervals. Variables were extracted from an ~18‐s window within the 20‐s plateau of each test contraction (excluding ~1 s at each end): (i) torque coefficient of variation (%), (ii) mean discharge rate, (iii) inter‐spike interval coefficient of variation (%) and (iv) intramuscular coherence metrics (see below). Motor unit recruitment threshold was defined as the torque (%MVT) at the first discharge. Coefficients of variation were calculated as the standard deviation of torque or inter‐spike interval divided by the mean over the window, expressed as a percentage.

Intramuscular coherence between motor unit spike trains was used to estimate common synaptic oscillations to the motoneuron pool [31, 33]. Coherence was computed between two cumulative spike trains (CSTs) formed by summing the binary discharge trains of randomly selected motor units. For each participant, each CST comprised five motor units [32], and the procedure was repeated for up to 100 random permutations to obtain pooled estimates, so coherence comparisons were unaffected by differences in the number of decomposed units. For each permutation, coherence was calculated between detrended CSTs using Welch's periodogram with a 1‐s Hanning window and 95% overlap [33]. Coherence spectra were averaged across permutations and transformed into *z*‐scores^S46^. Only *z*‐scores exceeding the bias threshold were retained, defined as the mean *z*‐coherence between 250 and 500 Hz, a frequency range with no expected physiological coherence [32, 33]. Differences in common synaptic input between conditions were quantified as the area under the curve (AUC) of the *z*‐coherence within the delta (δ, 1–5 Hz), alpha (α, 5–15 Hz) and beta (β, 15–35 Hz) bands (Figure 2). These bands are the frequency ranges most consistently examined in motor unit coherence studies and have been linked to different temporal features of common neural input to the motoneuron pool [11, 30, 33]. Delta‐band coherence is commonly associated with low‐frequency common input related to force fluctuations; alpha‐band coherence may include tremor‐related, spinal and afferent contributions; and beta‐band coherence is commonly associated with corticospinal input [11, 30, 33]. However, because coherence does not permit definitive source localisation, these bands were interpreted conservatively as indexing frequency‐specific organisation of common input rather than discrete anatomical generators. A plain language description of each neuromuscular outcome is presented in Supporting Information S1.

**FIGURE 2:**
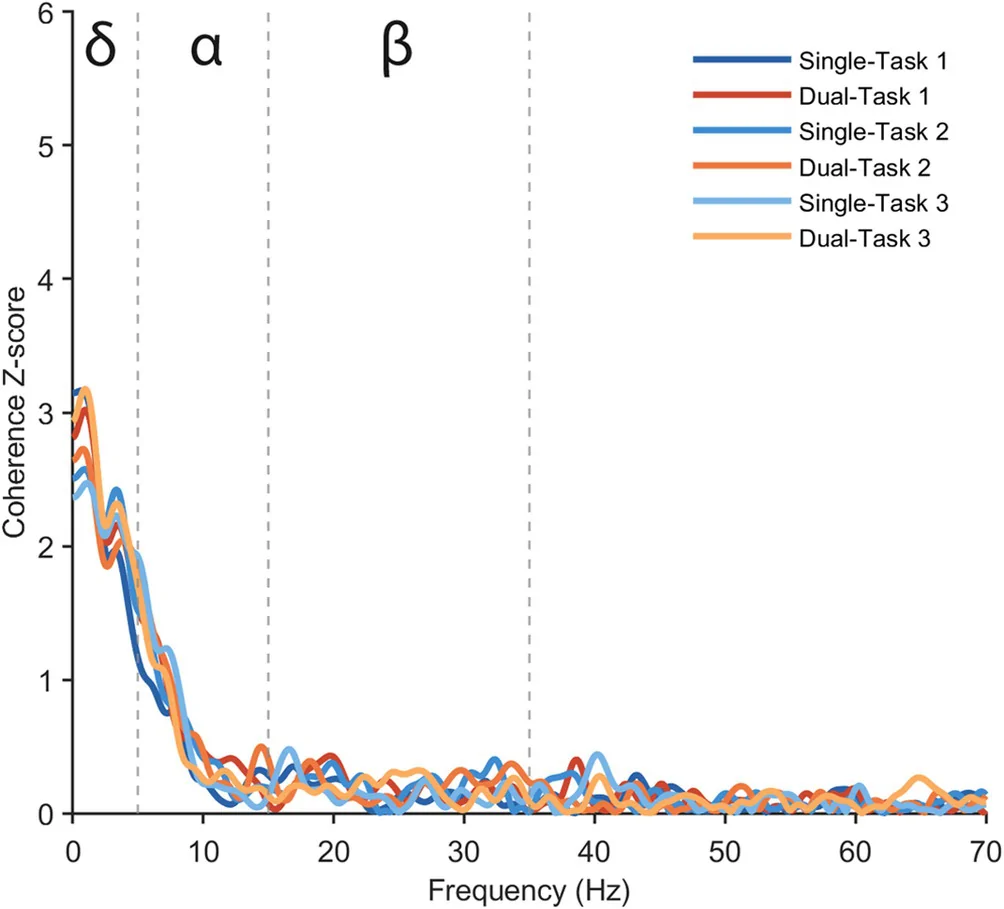
Coherence *Z*‐scores during single‐task and dual‐task isometric ankle dorsiflexion in a participant with sarcopenia. Coherence *Z*‐scores as a function of frequency (0–70 Hz) computed from the cumulative spike trains of motor units identified from the tibialis anterior during isometric contractions at 30% of maximal voluntary torque. Each line represents one trapezoidal contraction, with single‐task trials shown in blue shades (Single‐Task 1–3) and dual‐task trials shown in orange‐red shades (Dual‐Task 1–3). The dashed vertical lines delineate frequency bands of interest: delta (δ, 1–5 Hz), alpha (α, 5–15 Hz) and beta (β, 15–35 Hz).

### 2.5 Statistical Analysis

All analyses were conducted using robust linear mixed‐effects models to mitigate violations of classical linear model assumptions and downweigh the effects of outliers. Robust estimates were obtained via M‐estimation within linear mixed‐effects models. Primary inference emphasised point estimates and 95% confidence intervals (CIs) rather than null‐hypothesis significance testing.

Separate robust linear mixed‐effects models for force and motor unit–derived variables tested group (athletes, controls and sarcopenic) by condition (single and dual tasks) effects and their interaction, adjusting for sex (fixed effect) and motor unit recruitment threshold (covariate) when appropriate. Trials and motor units (for mean discharge rates and inter‐spike interval coefficient of variation) were nested within participants, and random intercepts were included to account for intraindividual correlation. The unbalanced group sizes do not bias these mixed‐effects models, which do not require balanced designs. Nesting of trials and motor units within participants provides the observation base, random intercepts capture within‐participant correlation and robust estimation limits outlier influence. CIs for the fixed‐effect coefficients were Wald‐type intervals based on the robust standard errors; for each contrast, bootstrap CIs were computed with 2000 resamples. Interpretation focused on the magnitude and precision of mean differences. Effects were considered noteworthy, and are described as significant, when their 95% CI excluded zero. The potential influence of age was evaluated by adding age to each primary model, modelling age against each outcome without group, quantifying age–group overlap and collinearity and conducting an exploratory 1:1 nearest‐neighbour age‐matched sarcopenic–control analysis. Because age adjustment did not alter the primary conclusions, age was omitted from the final models. Full sensitivity analyses are reported in Supporting Information S2. An exploratory analysis was conducted comparing subgroups of endurance‐ and power‐type athletes, reported in Supporting Information S3. Recruitment threshold analysis is reported in Supporting Information S4. As an additional exploratory analysis, we used previously published physical function and maximal strength data [24] to test whether the single‐ to dual‐task change in force CoV was associated with baseline maximal strength and functional performance (Supporting Information S5).

All analyses and figure generation were conducted using RStudio (version 2024.04.2) and MATLAB (Version 2023b, The MathWorks, Natick, MA, United States). The dataset, R code and packages information can be found at https://github.com/orssatto/mucoh‐sarcopenia.

## 3 Results

### 3.1 Participants and Motor Unit Yield

Fifty‐six participants (12 sarcopenic, 23 controls and 21 masters athletes) were initially tested. Four were excluded (one sarcopenic and one control unable to perform the dual task; two athletes withdrew due to time constraints). Eleven sarcopenic [mean ± standard deviation age, 80.1 ± 8.1 years (range 69–97), and body mass, 64.6 ± 13.1 kg, 7 females], 22 controls [74.8 ± 6.5 years (range 66–86), 70.7 ± 13.7 kg, 13 females] and 19 masters athletes [70.3 ± 6.1 years (range 65–86), 69.7 ± 15.0 kg, 6 females] were included in the analysis. A summary of the participants' characteristics is reported in Supporting Information S6, and the full cohort characterisation has been previously reported [24].

In total, 8302 motor unit spike trains were identified across contractions and conditions, derived from 251 motor units in participants with sarcopenia, 638 in controls and 735 in masters athletes. The median (first–third quartiles) number of motor units per participant was sarcopenic [females 18 (11–24), males 31 (27–34)], controls [females 22 (20–27), males 38 (31–44)] and masters athletes [females 27 (19–32), males 46 (41–50)].

### 3.2 Torque and High‐Density Electromyography Outcomes

Plateau torque remained close to 30% MVT in all groups during both single‐ and dual‐task contractions. There was no main effect of condition on plateau torque [β = −0.01% MVT, 95% CI (−0.18, 0.16), *t* = −0.08], or group × condition interaction [β = −0.01% MVT, 95% CI (−0.24, 0.22), *t* = −0.07]. Any between‐condition differences in motor unit discharge properties reported below therefore cannot be attributed to differences in the force produced.

Figures 3, 4, 5, 6, 7, 8 show estimated marginal means and mean differences for each variable.

**FIGURE 3:**
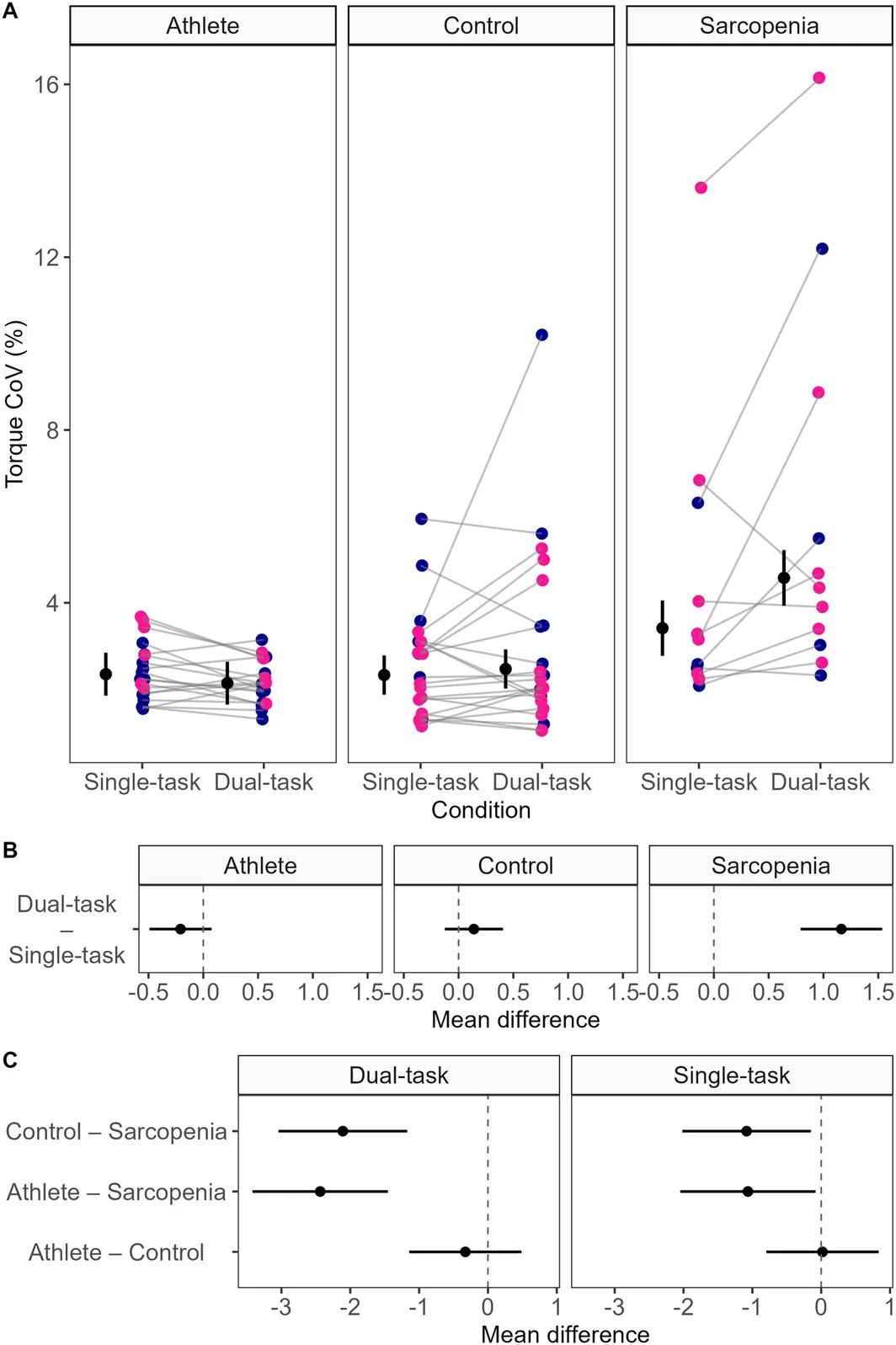
Effects of dual‐tasking on torque coefficient of variation (CoV) across groups. Panel A displays the participant‐level torque CoV during single‐task and dual‐task conditions for the athlete, control and sarcopenic groups. Each coloured point represents the mean torque CoV of one participant (blue = male, pink = female). The grey lines connect the same participant across conditions. Panel B displays the condition contrasts (dual‐task–single‐task) shown separately for each group. Torque CoV remained unchanged for athletes and controls and increased markedly for sarcopenic individuals under dual‐tasking. Panel C displays the group contrasts, shown separately for dual‐ and single‐task conditions. Under dual‐tasking, sarcopenic individuals exhibited significantly higher torque CoV compared with both athletes and controls. For all panels, black circles and error bars indicate estimated marginal means ± 95% confidence intervals.

**FIGURE 4:**
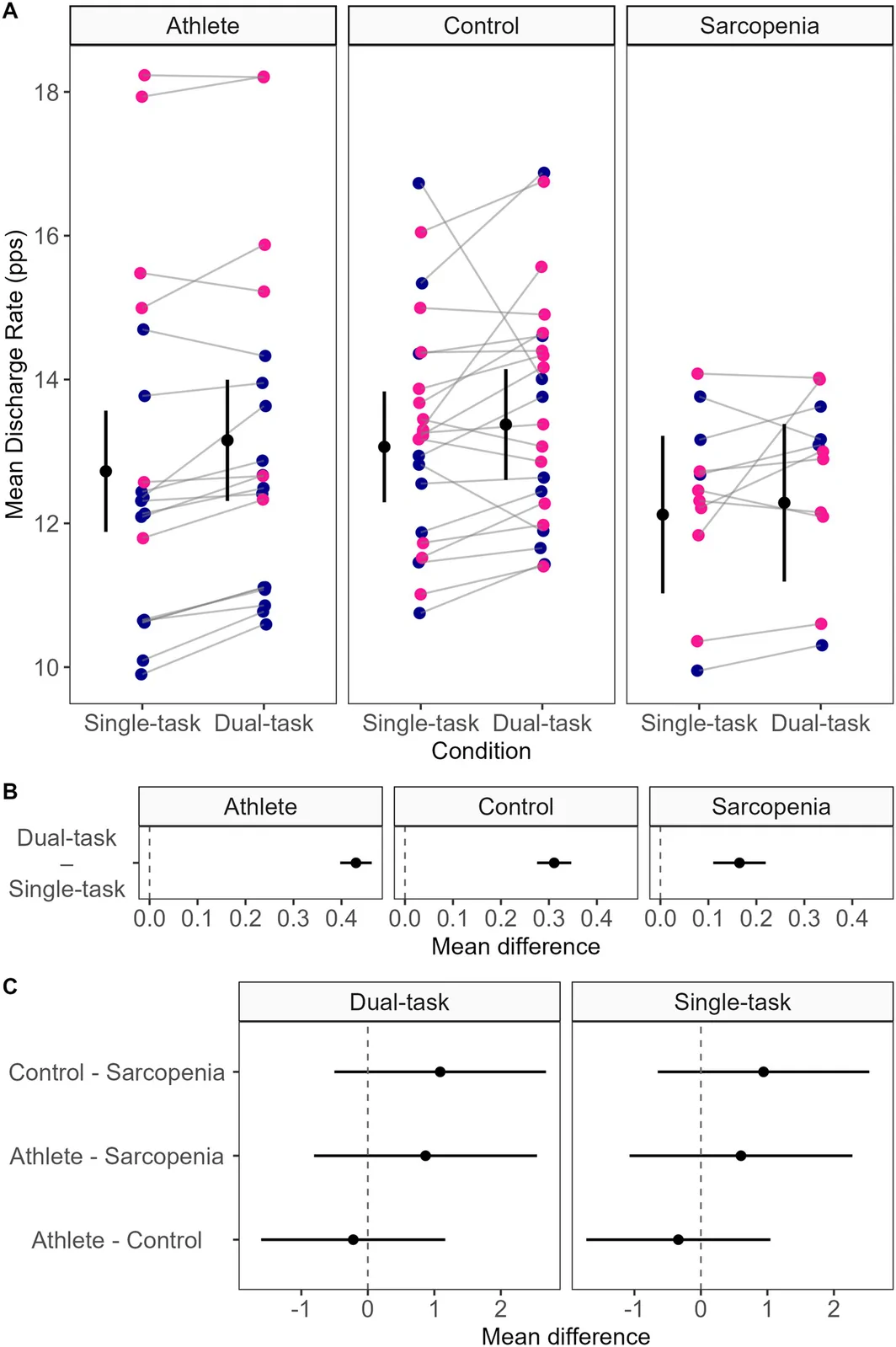
Effects of dual‐tasking on mean motor unit discharge rates across groups. Panel A displays the participant‐level mean discharge rates (pps) during single‐task and dual‐task conditions for the athlete, control, and sarcopenia groups. Each coloured point represents the mean discharge rate of one participant (blue = male, pink = female). The grey lines connect the same participant across conditions. Panel B displays the condition contrasts (dual‐task–single‐task) shown separately for each group. Mean discharge rates were significantly increased during dual‐tasking compared with single‐tasking across all groups. Panel C displays the group contrasts, shown separately for dual‐ and single‐task conditions. No significant group differences were detected in either condition. For all panels, black circles and error bars indicate estimated marginal means ± 95% confidence intervals.

**FIGURE 5:**
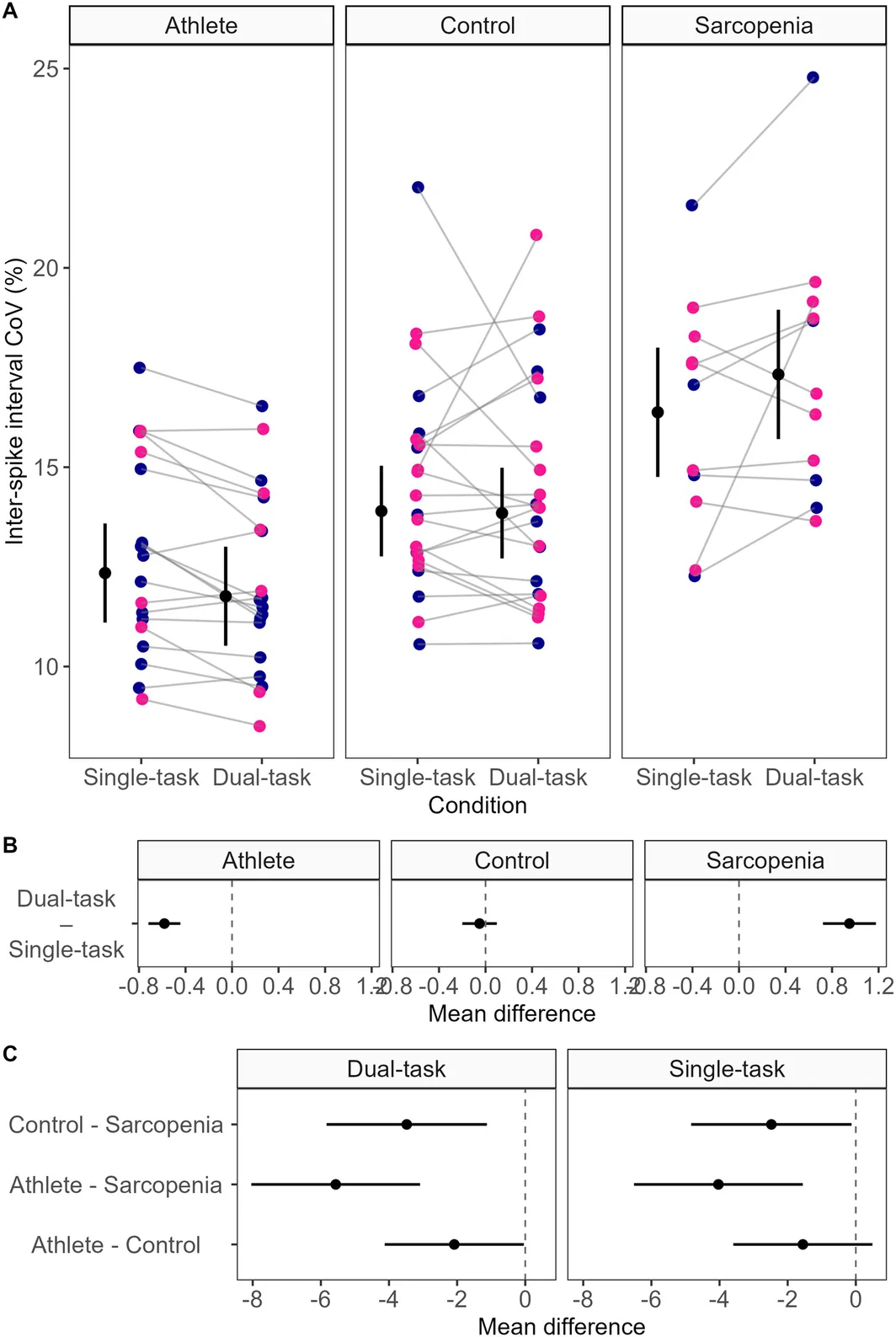
Effects of dual‐tasking on inter‐spike interval variability across groups. Panel A displays the participant‐level inter‐spike interval coefficient of variation (CoV, %) during single‐task and dual‐task conditions for the athlete, control, and sarcopenia groups. Each coloured point represents the mean value for one participant (blue = male, pink = female). The grey lines connect the same participant across conditions. Panel B displays the condition contrasts (dual‐task–single‐task) shown separately for each group. Inter‐spike interval CoV was significantly reduced in athletes, remained unchanged in controls and increased in sarcopenic individuals under dual‐tasking. Panel C displays the group contrasts, shown separately for dual‐ and single‐task conditions. Under dual‐tasking, sarcopenic individuals exhibited significantly higher inter‐spike interval CoV compared with both athletes and controls, whereas athletes demonstrated lower variability than controls. For all panels, the black circles and error bars indicate estimated marginal means ± 95% confidence intervals.

**FIGURE 6:**
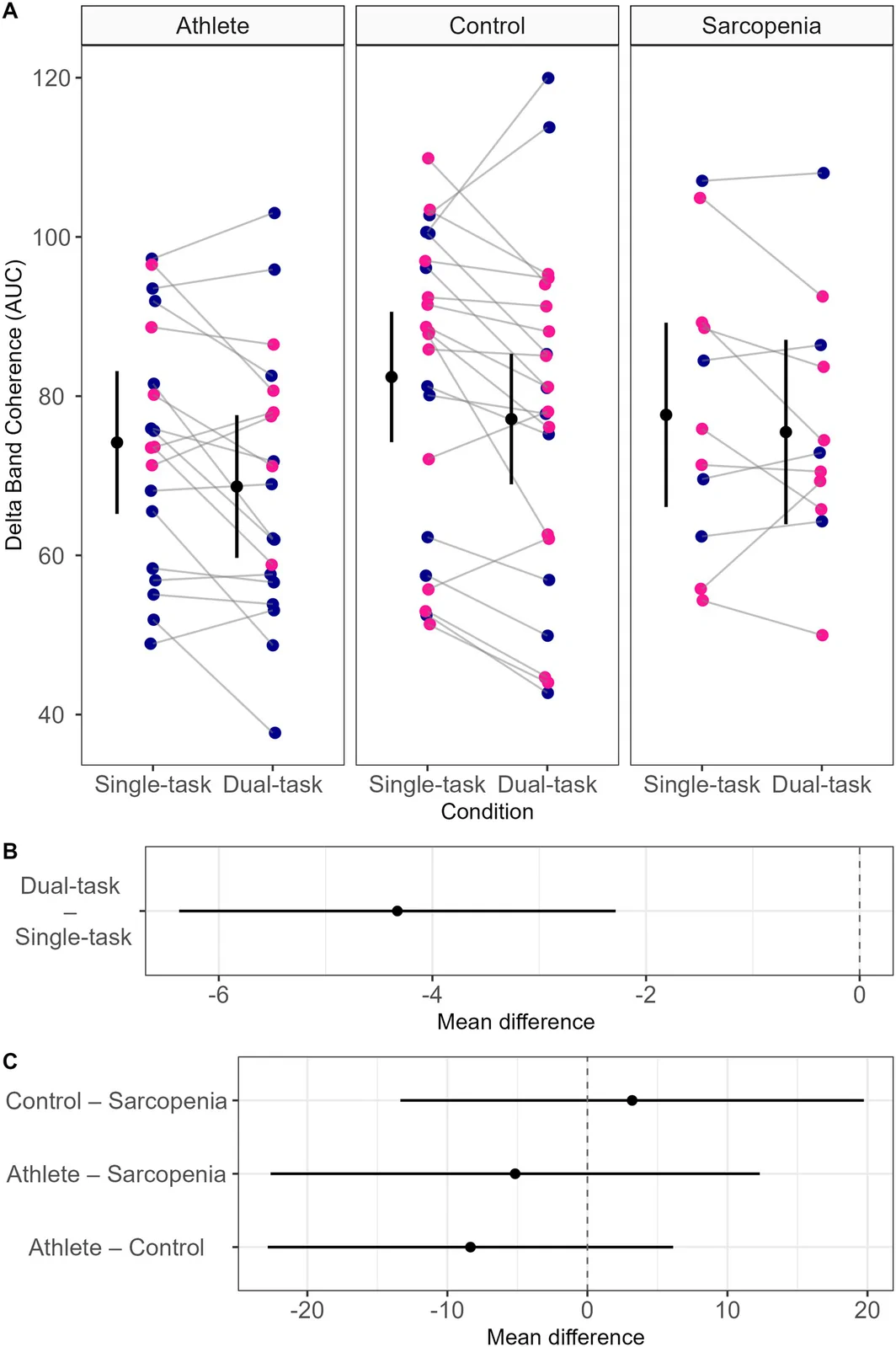
Effects of dual‐tasking on delta‐band coherence across groups. Panel A displays the participant‐level delta‐band coherence area under the curve (AUC) during single‐task and dual‐task conditions for the athlete, control, and sarcopenia groups. Each coloured point represents the mean value for one participant (blue = male, pink = female). Panel B displays the condition contrasts (dual‐task–single‐task), collapsed across groups. Delta‐band coherence was significantly reduced during dual‐tasking compared with single‐tasking across all groups. Panel C displays the group contrasts, collapsed across conditions. No significant differences were observed between groups in either condition. For all panels, the black circles and error bars indicate estimated marginal means ± 95% confidence intervals.

**FIGURE 7:**
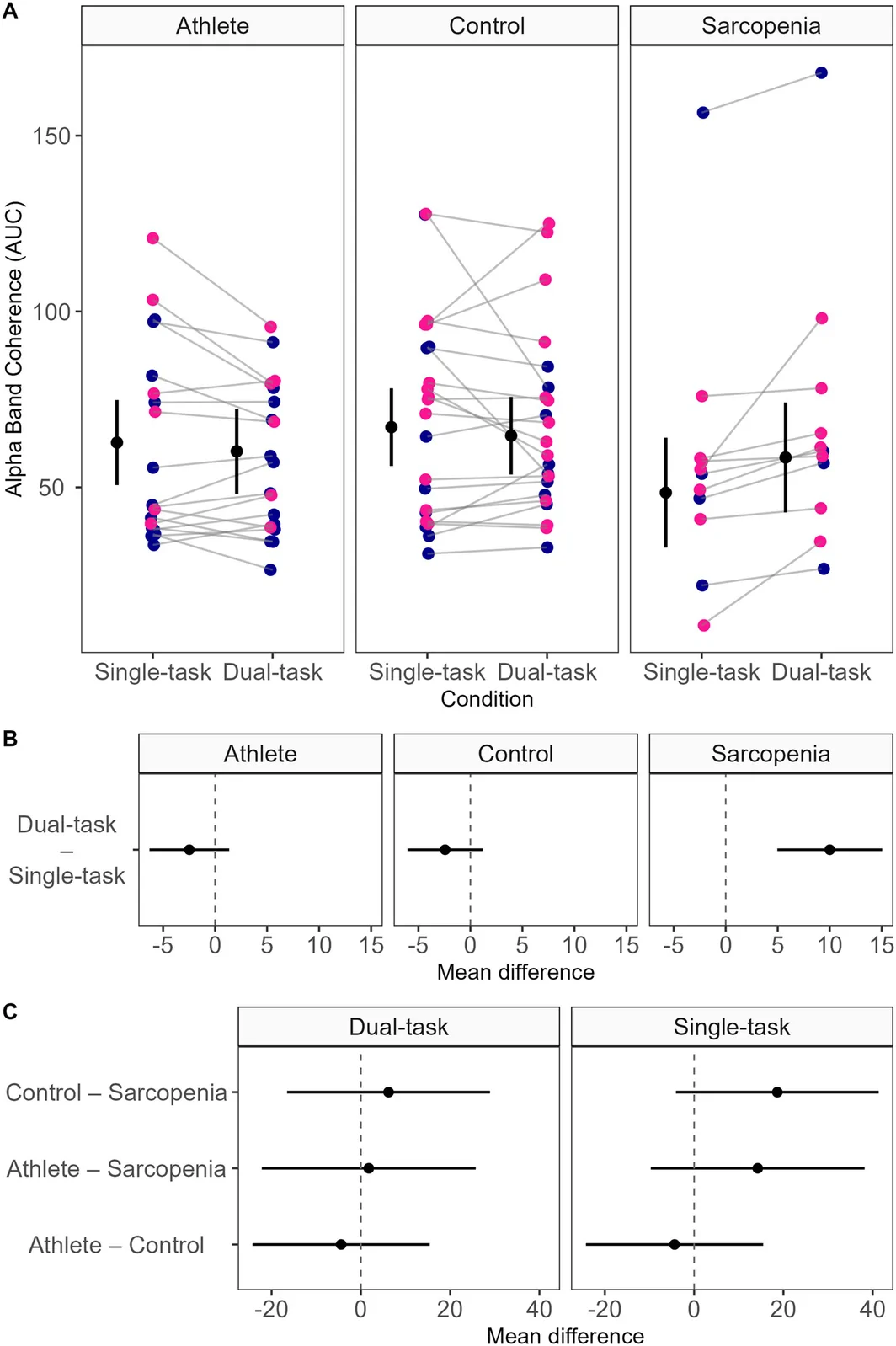
Effects of dual‐tasking on alpha‐band coherence across groups. Panel A displays the participant‐level alpha‐band coherence area under the curve (AUC) during single‐task and dual‐task conditions for the athlete, control, and sarcopenia groups. Each coloured point represents the mean value for one participant (blue = male, pink = female). Panel B displays the condition contrasts (dual‐task–single‐task) shown separately for each group. Alpha‐band coherence remained unchanged in athletes and controls but increased in sarcopenic individuals during dual‐tasking. Panel C displays the group contrasts, shown separately for dual‐ and single‐task conditions. No significant differences were observed between groups in either condition. For all panels, the black circles and error bars indicate estimated marginal means ± 95% confidence intervals.

**FIGURE 8:**
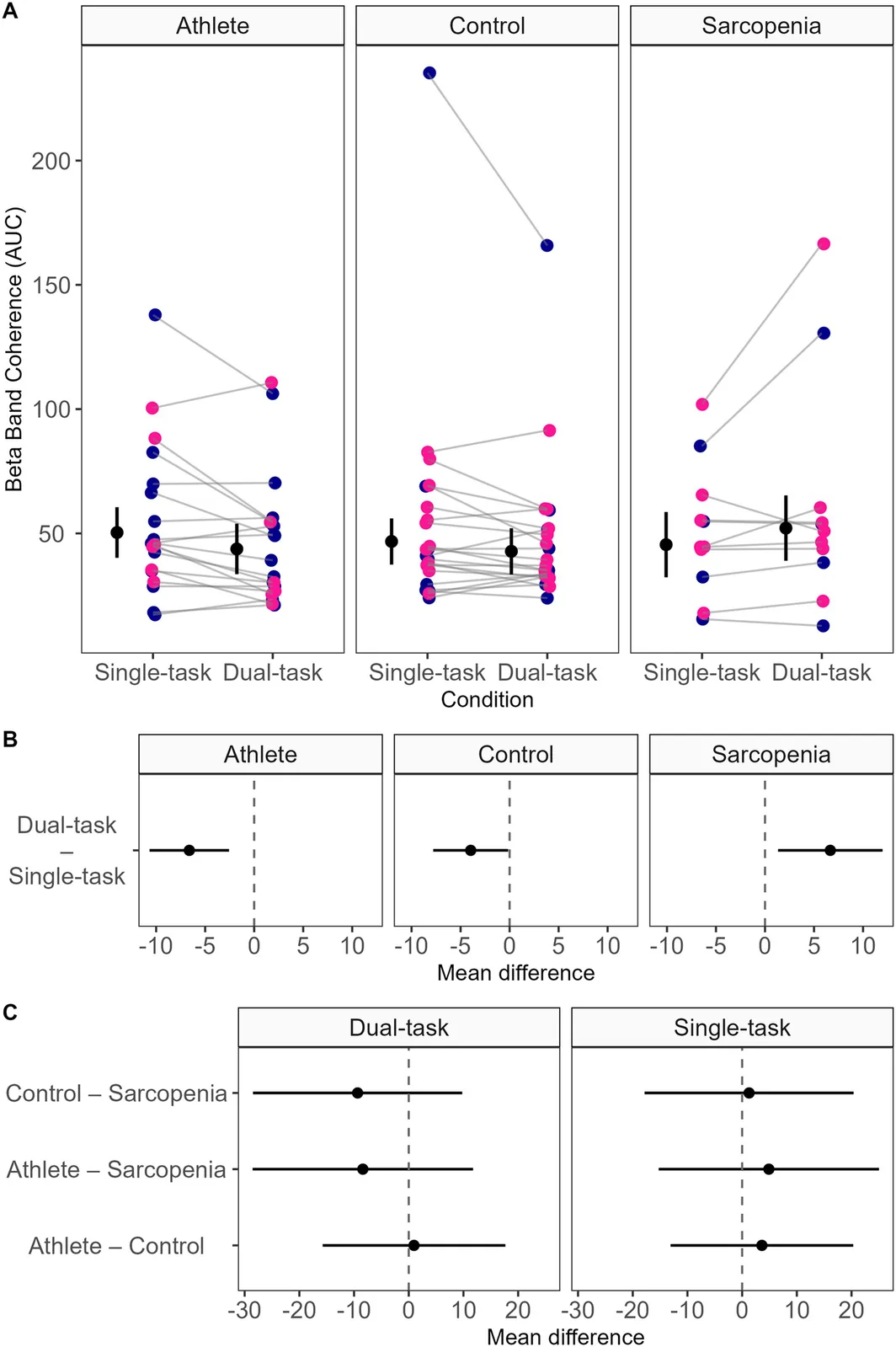
Effects of dual‐tasking on beta‐band coherence across groups. Panel A displays the participant‐level beta‐band coherence area under the curve (AUC) during single‐task and dual‐task conditions for the athlete, control, and sarcopenia groups. Each coloured point represents the mean value for one participant (blue = male, pink = female). Panel B displays the condition contrasts (dual‐task–single‐task) shown separately for each group. Beta‐band coherence was significantly reduced in athletes and controls under dual‐tasking but increased in sarcopenic individuals. Panel C displays the group contrasts, shown separately for dual‐ and single‐task conditions. No significant differences were observed between groups in either condition. For all panels, the black circles and error bars indicate estimated marginal means ± 95% confidence intervals.

Torque steadiness was reduced in sarcopenic and worsened further under the dual‐task condition. The model identified a group × condition interaction for torque coefficient of variation (a proxy of force steadiness) [β = −1.37 (−1.84, −0.91), *t* = −5.75]. Within each condition, the sarcopenic group was less steady (higher CoV) than both controls and athletes, which did not differ from one another (Figure 3C). From single‐ to dual‐task, CoV increased in the sarcopenic group only (Figure 3B). Sex had no effect [β = 0.06 (−0.52, 0.65), *t* = 0.20].

Mean discharge rates (a proxy of the neural drive to the muscle) increased in all groups during the dual‐task condition. The model returned a significant group × condition interaction term for mean discharge rate [β = 0.27 (0.20, 0.33), *t* = 8.16]; however, pairwise comparisons identified no group‐specific effect. Discharge rate increased comparably from single‐ to dual‐task in all three groups (Figure 4B), with no group differences within either condition (Figure 4C). Motor units recruited at higher thresholds discharged at lower rates [recruitment threshold: β = −0.031 (−0.034, −0.028), *t* = −20.0], and males discharged at lower rates than females [sex: β = −1.3 (−2.4, −0.3), *t* = −2.51].

Neural control steadiness worsened in sarcopenic, improved in athletes and remained unchanged in controls during the dual‐task condition. The model identified a group × condition interaction for inter‐spike interval CoV (a proxy of neural drive steadiness) [β = −1.53 (−1.80, −1.27), *t* = −11.3]. Within each condition, the sarcopenic group was less steady (higher CoV) than both controls and athletes (Figure 5C). From single‐ to dual‐task, CoV increased in the sarcopenic group, decreased in athletes and was unchanged in controls (Figure 5B); athletes were also lower than controls under dual‐task only. Higher threshold motor units showed greater variability [recruitment threshold: β = 0.14 (0.13, 0.15), *t* = 27.0]. There was no effect of sex [β = 0.02 (−1.51, 1.55), *t* = 0.03].

Common input associated with force fluctuations decreased in all groups during the dual‐task condition. For common neural input related to force fluctuations (delta‐band), the model identified a main effect of condition with no interaction [*β* = 5.53 (2.31, 8.75), *t* = 3.37]: Coherence decreased from single‐ to dual‐task in all groups, with no group differences in either condition (Figure 6). Sex had no effect [β = −3.04 (−13.9, 7.8), *t* = −0.55].

Involuntary physiological tremor–related fluctuations in common input increased in the sarcopenic group during the dual‐task condition. The model identified a group × condition interaction for alpha‐band coherence (common input associated with involuntary physiological tremor) [β = −12.5 (−18.8, −6.2), *t* = −3.86]. Coherence increased from single‐ to dual‐task in the sarcopenic group only, was unchanged in controls and athletes and did not differ between groups within either condition (Figure 7). Sex had no effect [β = −13.0 (−27.7, 1.7), *t* = −1.74].

Corticospinal coupling was increased in the sarcopenic group and reduced in controls and athletes during the dual‐task condition. The model identified a group × condition interaction for beta‐band coherence (corticospinal contribution to common input) [β = −13.3 (−20.0, −6.6), *t* = −3.89]. From single‐ to dual‐task, coherence increased in the sarcopenic group but decreased in controls and athletes (Figure 8). Sex had no effect [β = −3.7 (−15.9, 8.6), *t* = −0.59].

### 3.3 Exploratory Analyses

Age does not explain the phenotype‐specific dual‐task responses. In models omitting group, older age was associated with modestly greater variability of torque and inter‐spike interval, but not with discharge rate or coherence. Adding age to the primary models revealed no age effect and did not change the group or dual‐task findings, and an age‐matched comparison retained the key sarcopenic‐specific effects (Supporting Information S2).

Dual‐task responses are broadly similar between endurance‐ and power‐type athletes. Torque steadiness did not differ between endurance‐type (*n* = 10) and power‐type (*n* = 9) athletes, and both showed increased discharge rate and reduced inter‐spike interval variability under dual‐tasking, albeit higher in power athletes. Coherence responses were subgroup‐specific and exploratory given the small samples (Supporting Information S3).

The dual‐task increase in force unsteadiness relates to function more than to strength. Using physical function and maximal strength from our companion study [24], we found that a larger dual‐task increase in torque CoV was associated with poorer baseline functional power and mobility, but not with maximal dorsiflexion strength. This suggests vulnerability of force steadiness to cognitive loading relates more to broad functional capacity than to local strength (Supporting Information S5).

## 4 Discussion

This study examined whether and how force steadiness differed during single‐ and dual‐task submaximal contractions in older adults with sarcopenia compared with non‐sarcopenic controls and masters athletes. We distinguish torque coefficient of variation (steadiness of mechanical output), inter‐spike interval coefficient of variation (regularity of discharge, i.e., neural control steadiness) and intramuscular coherence, which reflects the frequency‐specific organisation of common synaptic input. Three main findings emerged. First, older adults with sarcopenia were less able to control torque steadily, which became worse when attention was divided by a cognitive task. In contrast, controls and masters athletes maintained stable performance. Second, this torque unsteadiness in individuals with sarcopenia was accompanied by greater inter‐spike interval variability, indicating that motoneuron discharge timing became less regular under cognitive load. Masters athletes showed the opposite pattern, with better neural drive steadiness during dual‐tasking, suggesting greater resilience of neuromuscular control. Third, dual‐tasking changed the common synaptic input to motoneurons in different ways across groups. All groups showed lower delta‐band coherence under cognitive load, indicating that a reduction in the common drive related to force fluctuations does not explain the different torque steadiness responses during dual task across groups. However, under cognitive load, the sarcopenic group showed increased alpha‐band coherence, consistent with a greater contribution of oscillatory common input that may include tremor‐related, spinal and afferent components [11, 31, 37]. This increase coincided with greater inter‐spike interval and torque variability and may have contributed to poorer force steadiness, although the present analyses do not establish a causal pathway between these outcomes. Masters athletes showed the opposite. They were the only group whose discharge regularity improved under cognitive load, and they showed no rise in alpha‐band oscillations together with the largest reduction in beta‐band coherence, consistent with different frequency‐specific modulation of their common synaptic input when attention was divided. These findings suggest that dual‐task performance in older adults depends not simply on muscle strength but on the steadiness and adaptability of neural drive to muscle, which is degraded in individuals with sarcopenia and preserved, or even enhanced, in lifelong athletes.

### 4.1 Neural Control Unsteadiness Accompanying Force Unsteadiness in Sarcopenia

Force steadiness during low‐force contractions depends on the temporal regularity of motor unit discharge. Increased inter‐spike interval variability has been linked to larger force fluctuations in older adults, particularly under cognitively demanding conditions [8]. Inter‐spike interval coefficient of variation is an index of discharge regularity linked to force steadiness, though it does not separate common from independent variability or provide frequency information. We therefore complemented it with coherence, making the two measures complementary rather than redundant. In this study, participants with sarcopenia exhibited greater discharge variability during single‐task contractions, which was amplified with dual‐tasking, paralleling the marked deterioration in torque steadiness. Mean motor‐unit discharge rate increased similarly across groups during dual‐tasking, indicating that the group‐specific changes in steadiness were not explained by differences in the average firing rate of the sampled motor units. Rather, the key abnormality in individuals with sarcopenia appears to lie in the unsteadiness of motor unit discharge timing. This distinction is important because it suggests that the deterioration under cognitive stress was explained not by mean motor‐unit discharge rate but by a reduced ability to organise that drive consistently enough to maintain steady force.

Dividing attentional resources is known to alter corticospinal excitability and reduce the precision of sensorimotor integration, potentially increasing variability in descending and spinal synaptic input in older adults [12, 13]. In the context of sarcopenia, where motor unit remodelling, reduced motoneuron number and excitability and impaired neuromuscular junction transmission have all been reported [4, 24, 38], the motoneuron pool may operate closer to its functional limits. Including an additional cognitive demand may therefore expose instability that is partly compensated during isolated motor tasks. This may help explain why participants with sarcopenia showed a clear deterioration in force steadiness during dual‐tasking whereas controls did not, despite the same general increase in mean discharge rate. Masters athletes provide an informative contrast because they appear to preserve a greater capacity to maintain stable motor output when attention is divided. In our study, athletes not only preserved torque steadiness but also reduced motor unit discharge variability during dual‐tasking. Lifelong training is typically performed in dynamic environments that demand ongoing integration of sensory information, attentional shifting, decision‐making and motor execution [22, 23]. Such repeated exposure may promote more efficient allocation of central resources and more adaptable control strategies [22, 23]. Age may contribute generally to motor unsteadiness, as indicated by the group‐free models, but does not explain the phenotype‐specific dual‐task responses. The central findings in individuals with sarcopenia persisted after age adjustment and exploratory age matching (Supporting Information S2). Nevertheless, age, training history and health differed across phenotypes, so residual confounding remains possible [8].

### 4.2 Frequency‐Specific Alterations in Common Synaptic Input

Coherence analysis between motor units provided insight into how common synaptic input was modulated under cognitive load. Notably, there were no significant between‐group differences in delta‐, alpha‐, or beta‐band coherence during single‐task contractions. Thus, the groups were not distinguished by large baseline differences in frequency‐specific common input during the isolated motor task. Instead, the divergence emerged in how each phenotype adapted when cognitive demand was superimposed on force production. Delta‐band coherence decreased in all groups during dual‐tasking, suggesting that low‐frequency common input was globally modulated when attentional resources were divided. This may seem counterintuitive, as low‐frequency common input is closely related to force fluctuations [11]. However, the delta‐band reduction occurred in all three groups, whereas torque steadiness deteriorated only in sarcopenia. The delta‐band change therefore does not parallel the sarcopenia‐specific loss of steadiness and may instead reflect a broad redistribution of neural control under cognitive load rather than a phenotype‐specific abnormality. One possible explanation is that the addition of a cognitive demand may introduce an additional source of oscillation to the common synaptic input to the motoneuron pool, which may reduce the relative contribution of the original low‐frequency input. This interpretation is consistent with previous evidence showing that when independent common inputs are introduced to a motoneuron pool, they can decorrelate motor unit activity within specific frequency bands [37]. In contrast, alpha‐band coherence showed differential dual‐task responses across phenotypes. Alpha‐band oscillations are commonly associated with physiological tremor and represent components of the neural input that are not directly related to the intended force output. Because these oscillations can still be expressed in the force signal^S47^, they contribute to unwanted fluctuations in force. Thus, they can be interpreted as a form of common ‘noise’ in the neural drive that reduces the accuracy of force production [11]. This is consistent with alpha‐band oscillations reflecting, in part, spinal contributions modulated by afferent feedback related to muscle length and stiffness, complementing the neural filtering of physiological tremor described previously [33]. These observations are consistent with a spinal/afferent contribution but do not identify a specific source [39]. In this study, the selective increase in alpha‐band coherence in the sarcopenic group during dual‐tasking provides a likely explanation for their increased motor unit discharge variability and greater torque variability under cognitive load. Despite the reduction in delta‐band coherence observed in all groups, only individuals with sarcopenia showed an increase in these common ‘noise’ oscillations, suggesting that their neural control becomes less effective at maintaining stable force when cognitive demands are increased. Beta‐band coherence further highlighted differences between groups. Beta‐band oscillations have been widely linked to corticospinal coupling and are thought to reflect the contribution of cortical input to the motoneuron pool [11]. Beta‐band coherence increased in individuals with sarcopenia during dual‐tasking, which may indicate a greater reliance on cortical input when cognitive demands are increased. Conversely, the reduction in beta‐band coherence observed in controls and, more prominently, in masters athletes suggests a more adaptable regulation of common synaptic input. Although the present design does not permit causal inference, the emergence of these opposite modulation patterns only under dual‐task conditions supports the idea that impaired neural adaptability, rather than simply impaired strength, may distinguish sarcopenic from more resilient ageing phenotypes. Because coherence can depend on discharge rate, we considered whether the higher rate under dual‐tasking could be an artefact. Two features argue against this: CSTs comprised a fixed five units, and discharge rate rose only ~2.6% and similarly in all groups, whereas the coherence changes were group‐specific. The small and broadly similar increase in discharge rate across groups is therefore unlikely to fully account for the group‐ and frequency‐specific coherence responses.

### 4.3 Clinical Implications

The clinical importance of these findings is that sarcopenia may involve deficits in neuromuscular control that are not fully captured by measures of muscle mass or maximal strength alone [7]. Force steadiness has been linked to mobility performance, postural control and other clinically meaningful aspects of function [40], and dual‐task performance is strongly connected to fall risk and loss of independence in older adults [17]. Consistent with this, in our cohort, the dual‐task increase in force CoV was related to poorer functional power and mobility but not to maximal dorsiflexion strength (Supporting Information S5). In that context, the findings from this study suggest that traditional single‐task strength testing may underestimate vulnerability in individuals with sarcopenia when they are required to divide attention, which is precisely how many real‐world activities are performed.

The findings from this study also have implications for intervention design. Resistance training is recommended to improve muscle strength in people with sarcopenia^S48^, but strategies that enhance the stability and adaptability of neural drive may also be valuable. Challenging balance training, multicomponent programmes, dual‐task training and exercise modalities that require ongoing sensorimotor integration may be especially relevant if the goal is not only to increase force capacity but also to improve the ability to control force under cognitively demanding conditions. In fact, dual‐task exercises are known to improve dynamic balance and functional mobility and reduce fall rates in older adults^S49^. The preserved discharge regularity and flexible modulation of common synaptic input observed in masters athletes further support the concept that neural adaptability is modifiable and may represent a meaningful therapeutic target.

Force and motor unit discharge variability under cognitive load may prove useful as clinical and mechanistic markers of neuromotor vulnerability in older adults with sarcopenia. Longitudinal studies are now needed to determine whether these physiological features predict mobility decline, falls or loss of independence and whether interventions that improve discharge regularity also lead to clinically meaningful functional outcomes. This study provides a mechanistic basis for moving beyond a purely muscle‐centric view of sarcopenia towards a broader model that more fully incorporates neural control [7].

### 4.4 Strengths and Limitations

This study has several strengths. The use of HD‐EMG combined with motor unit decomposition allowed high‐resolution, noninvasive characterisation of motor unit discharge behaviour and common synaptic input in vivo. Analysing discharge variability at the motor unit level provides mechanistic insight beyond traditional surface EMG amplitude measures. The inclusion of three distinct ageing phenotypes enabled examination of both vulnerability and resilience within older adults, thereby strengthening interpretation of phenotype‐specific adaptations. However, several important limitations warrant consideration. First, the cross‐sectional design precludes causal inference regarding whether altered discharge variability or coherence contributes directly to functional decline or instead reflects adaptation. Second, the sarcopenic group was relatively small, which limits precision and warrants caution when interpreting interaction‐heavy analyses, particularly for coherence outcomes. Although age was associated with torque and inter‐spike interval variability in models excluding group, age adjustment did not alter the primary findings and an exploratory matched analysis retained the central sarcopenic‐specific dual‐task effects (Supporting Information S2). Nevertheless, the groups were not prospectively age‐matched, and residual confounding remains possible. Third, neuromuscular assessments were performed during submaximal isometric dorsiflexion of tibialis anterior; although this muscle is relevant to gait and postural control and is well suited to HD‐EMG decomposition, the findings may not generalise to other muscle groups or to more dynamic tasks. Fourth, cognitive‐task accuracy, response rate and formal cognitive screening were not recorded. Therefore, although all included participants met the prespecified task‐continuation criteria, we could not quantify cognitive‐task performance or determine whether the concurrent serial subtraction imposed an equivalent cognitive demand across groups. Randomised alternation of conditions reduced potential order and fatigue effects but did not control for differences in cognitive demand. Finally, because this study was conducted in individuals with sarcopenia diagnosed using the SDOC definition [2], the results may not generalise to individuals classified as sarcopenic under other diagnostic definitions.

## 5 Conclusions

This study shows that older adults with sarcopenia had poorer force steadiness and greater motor unit discharge rate variability (inter‐spike interval CoV, i.e., a proxy of neural control unsteadiness) and that both deficits worsened when a cognitive task was added. This pattern was not seen in healthy controls or masters athletes. Mean motor‐unit discharge rate increased similarly across all groups, suggesting that the deterioration in individuals with sarcopenia was not explained by the average firing rate of the sampled motor units but was instead characterised by less regular discharge timing. Group differences in the modulation of common synaptic input further suggest that ageing phenotypes differ in how the nervous system adapts when attentional demands increase. Masters athletes maintained more regular motor unit discharge and showed more flexible regulation of common synaptic input during dual‐tasking. These findings suggest that greater motor unit discharge variability during cognitive challenge may represent a neural feature of functional impairment in sarcopenia and support the inclusion of neuromuscular control measures, in addition to muscle mass and maximal strength, in clinical evaluation and intervention strategies.

## Funding

This study was supported by Deakin University.

## Conflicts of Interest

The authors declare no conflicts of interest.

## Supporting information


**DATA S1:** Supporting Information.

**DATA S2:** Supporting Information.

**DATA S3:** Supporting Information.

**DATA S4:** Supporting Information.

**DATA S5:** Supporting Information.

**DATA S6:** Supporting Information.

**DATA S7:** Supporting Information.

## Data Availability

The data that support the findings of this study are openly available in mucoh‐sarcopenia at https://github.com/orssatto/mucoh‐sarcopenia.
