# Supplementary material for: Dual‐Tasking Exacerbates Force and Neural Control Unsteadiness in Older Adults With Sarcopenia
Source: J Cachexia Sarcopenia Muscle. 2026 Oct 5;17(5):e70397. doi: 10.1002/jcsm.70397 (PMC13638254; doi:10.1002/jcsm.70397)
Supplement: Supplementary file 1 [file JCSM-17-e70397-s001.docx]

Supplementary Material 6. Included participants’ characteristics

**Table S6.** Participant characteristics, physical activity, physical function and body composition of older adults with sarcopenia, nonsarcopenic controls and masters athletes.

| **Characteristic** | **Sarcopenic (n = 11)** | | **Controls (n = 22)** | | **Athletes (n = 19)** | |
| --- | --- | --- | --- | --- | --- | --- |
|  | **Mean ± SD** | **Range** | **Mean ± SD** | **Range** | **Mean ± SD** | **Range** |
| Age, years | 80.1 ± 8.1 | 69.0–97.0 | 74.8 ± 6.5 | 66.0–86.0 | 70.3 ± 5.1 | 65.0–86.0 |
| Height, m | 1.62 ± 0.10 | 1.49–1.78 | 1.66 ± 0.09 | 1.51–1.83 | 1.72 ± 0.11 | 1.44–1.92 |
| Body mass, kg | 64.6 ± 13.1 | 44.2–87.3 | 70.7 ± 13.7 | 46.0–101.5 | 69.7 ± 15.0 | 42.0–98.9 |
| Body mass index, kg·m⁻² | 24.7 ± 4.9 | 14.6–28.8 | 25.7 ± 4.3 | 18.7–36.4 | 23.3 ± 3.0 | 19.4–29.2 |
| Handgrip strength, kg | 21.5 ± 8.1 | 8.7–34.7 | 31.4 ± 10.3 | 17.6–52.3 | 37.6 ± 9.5 | 20.9–56.0 |
| Usual gait speed, m·s⁻¹ | 0.76 ± 0.13 | 0.39–0.87 | 1.38 ± 0.19 | 0.99–1.73 | 1.41 ± 0.18 | 1.07–1.83 |
| Fast gait speed, m·s⁻¹ | 1.02 ± 0.18 | 0.52–1.17 | 1.93 ± 0.33 | 1.18–2.67 | 2.16 ± 0.24 | 1.77–2.68 |
| Four-Square Step Test, s | 13.26 ± 4.35 | 9.58–24.35 | 8.79 ± 1.30 | 5.90–10.69 | 7.40 ± 0.76 | 6.15–9.08 |
| Timed Up and Go, s | 13.13 ± 5.07 | 9.43–27.71 | 6.87 ± 1.08 | 5.50–10.15 | 5.87 ± 0.65 | 4.56–7.50 |
| Five-times sit-to-stand, s | 17.06 ± 2.16 | 14.98–23.11 | 11.07 ± 1.49 | 8.85–14.25 | 9.56 ± 1.33 | 6.86–12.41 |
| Sit-to-stand power, W·kg⁻¹ | 1.90 ± 0.37 | 1.13–2.37 | 3.05 ± 0.48 | 2.35–4.19 | 3.83 ± 0.57 | 2.94–4.81 |
| Dorsiflexion peak torque, N·m | 10.8 ± 4.0 | 4.1–16.2 | 26.0 ± 7.0 | 14.4–40.6 | 27.5 ± 8.6 | 11.3–41.7 |
| Dorsiflexion peak torque/body mass, N·m·kg⁻¹ | 0.17 ± 0.07 | 0.07–0.30 | 0.37 ± 0.09 | 0.22–0.52 | 0.39 ± 0.09 | 0.22–0.56 |

Note. Participants in the present study were recruited as part of the same data collection described previously [PMID: 41292210]. Of the 56 older adults originally enrolled, 52 (11 sarcopenic, 22 nonsarcopenic controls and 19 masters athletes) were retained for the present analyses; four were excluded. All characterisation, strength, power and physical function measures reported here were obtained during the same testing session as those reported in that earlier work. The two datasets therefore share participants and cannot be treated as statistically independent samples.
