# Supplementary material for: Dual‐Tasking Exacerbates Force and Neural Control Unsteadiness in Older Adults With Sarcopenia
Source: J Cachexia Sarcopenia Muscle. 2026 Oct 5;17(5):e70397. doi: 10.1002/jcsm.70397 (PMC13638254; doi:10.1002/jcsm.70397)
Supplement: Supplementary file 2 [file JCSM-17-e70397-s002.docx]

Supplementary Material 4. Recruitment Thresholds

**Statistical analysis description**

Recruitment threshold was analysed using a robust linear mixed-effects model consistent with the models used for the other motor-unit outcomes. The model included sex, group, condition, and the group × condition interaction as fixed effects, with random intercepts for motor units nested within participants.

**Results**

Motor units were recruited at lower thresholds in sarcopenic, while dual-tasking produced small and group-specific shifts. The model identified a group × condition interaction for recruitment threshold, driven by the control × condition term [β = 0.69 (0.42, 0.95), t = 5.08]. From single- to dual-task, recruitment threshold increased in athletes and participants with sarcopenia, but decreased in controls (Table S4A and S4B). Within the single-task condition, the sarcopenic group had lower recruitment thresholds than both controls and athletes; under dual-task, recruitment threshold remained lower than in athletes but did not differ from controls (Table S4A and S4C). Athletes and controls did not differ within either condition. Sex had no effect [β = −1.90 (−4.18, 0.39), t = −1.62].

Table S4A. Estimated marginal means for motor unit recruitment threshold

| **Group** | **Condition** | **Estimated marginal mean** | **95% CI** |
| --- | --- | --- | --- |
| Athlete | Dual-task | 15.70 | 13.87 to 17.53 |
| Control | Dual-task | 14.66 | 12.96 to 16.36 |
| Sarcopenic | Dual-task | 11.35 | 8.85 to 13.85 |
| Athlete | Single-task | 15.39 | 13.56 to 17.22 |
| Control | Single-task | 15.03 | 13.33 to 16.73 |
| Sarcopenic | Single-task | 11.01 | 8.51 to 13.51 |

Table S4B. Condition contrasts per group.

| **Group** | **Contrast** | **Estimated difference** | **95% CI** |
| --- | --- | --- | --- |
| Athlete | Dual-task − Single-task | 0.31 | 0.13 to 0.49 |
| Control | Dual-task − Single-task | −0.37 | −0.57 to −0.18 |
| Sarcopenic | Dual-task − Single-task | 0.34 | 0.04 to 0.64 |

Table S4C. Group contrasts per condition

| **Condition** | **Contrast** | **Estimated difference** | **95% CI** |
| --- | --- | --- | --- |
| Dual-task | Athlete − Control | 1.05 | −1.97 to 4.07 |
| Dual-task | Athlete − Sarcopenic | 4.35 | 0.61 to 8.10 |
| Dual-task | Control − Sarcopenic | 3.31 | −0.29 to 6.91 |
| Single-task | Athlete – Control | 0.36 | −2.66 to 3.38 |
| Single-task | Athlete − Sarcopenic | 4.38 | 0.63 to 8.13 |
| Single-task | Control − Sarcopenic | 4.02 | 0.42 to 7.62 |

*Notes. Recruitment threshold is expressed relative to maximal voluntary torque (% MVT).*
