# Supplementary material for: Dual‐Tasking Exacerbates Force and Neural Control Unsteadiness in Older Adults With Sarcopenia
Source: J Cachexia Sarcopenia Muscle. 2026 Oct 5;17(5):e70397. doi: 10.1002/jcsm.70397 (PMC13638254; doi:10.1002/jcsm.70397)
Supplement: Supplementary file 3 [file JCSM-17-e70397-s003.docx]

Supplementary Material 5. Functional correlates of dual-task-related changes in force steadiness

**Summary**

Several measures of poorer functional performance were associated with a larger increase in force coefficient of variation (CoV) from single- to dual-task conditions, whereas local maximal dorsiflexion strength was not clearly associated with this change. These findings suggest that vulnerability of force steadiness to cognitive loading is more closely related to broader mobility and functional power than to maximal dorsiflexion torque alone.

All strength and functional measures used in these analyses (dorsiflexion peak torque, five-times sit-to-stand power, Timed Up and Go, usual and fast gait speed, and the Four-Square Step Test) were assessed in the same participants and reported in full in our companion study (PMID: 41292210). They were not measured or reported as new data in the present study.

**Statistical analysis description**

Force CoV was averaged across contractions for each participant within the single- and dual-task conditions. Participant-level change was calculated as mean dual-task CoV minus mean single-task CoV (positive values indicate a worsening of force steadiness during dual-tasking). Separate robust linear regression models were fitted for absolute dorsiflexion peak torque, peak torque normalised to body mass, five-times sit-to-stand power normalised to body mass, Timed Up and Go, usual gait speed, fast gait speed, and Four-Square Step Test performance. The primary models included each predictor and sex. Predictors were standardised and directionally recoded so that higher standardised values consistently represented poorer function. Associations are reported as the change in force CoV, in percentage points, per 1-SD poorer function, with 95% confidence intervals. Robust R² was reported for each full model, and incremental robust R² was calculated relative to a sex-only model. Secondary models included a predictor-by-sex interaction to estimate sex-specific slopes and male-female slope differences. No p-values were used for interpretation. These cohort-wide analyses did not adjust for phenotype group; consequently, estimated associations may partly reflect between-phenotype differences.

**Results**

Poorer functional performance was associated with a larger dual-task-related increase in force variability for several outcomes (Figure S5; Tables S5A and S5B). Each 1-SD worsening in Timed Up and Go was associated with a 0.442-percentage-point larger increase in force CoV [95% CI 0.212 to 0.672], while each 1-SD reduction in five-times sit-to-stand power was associated with a 0.484-percentage-point larger increase [95% CI 0.181 to 0.787]. Timed Up and Go and sit-to-stand power provided the largest incremental robust R² beyond sex alone (0.210 and 0.158, respectively).

Slower usual and fast gait speeds were also associated with larger dual-task increases in force CoV, although these estimates were smaller and less precise [usual gait speed: β = 0.292, 95% CI 0.008 to 0.576; fast gait speed: β = 0.311, 95% CI 0.022 to 0.600]. Their incremental robust R² values were 0.071 and 0.078, respectively. Four-Square Step Test performance was not clearly associated with the dual-task change [β = 0.161, 95% CI −0.081 to 0.403]. Neither absolute dorsiflexion peak torque [β = 0.198 per 1-SD poorer strength, 95% CI −0.155 to 0.551] nor torque normalised to body mass [β = 0.209, 95% CI −0.091 to 0.510] was clearly associated with the dual-task change, and their incremental robust R² values were small (0.023 and 0.035). Thus, dual-task deterioration in force steadiness was more closely related to broader functional power and mobility than to local maximal dorsiflexion strength.

**Figure S5.** Associations between poorer function and the single-to-dual-task change in force coefficient of variation. Points show sex-adjusted robust regression slopes and horizontal bars show 95% confidence intervals. Predictors were standardised and directionally recoded so that positive estimates consistently indicate that poorer function was associated with a larger increase in force CoV during dual-tasking. Each estimate is from a separate model.

Table S5A. Sex-adjusted associations between strength or functional outcomes and the single-to-dual-task change in force CoV.

| **Outcome** | **n** | **Original-unit slope** | **95% CI** | **Slope per 1 SD poorer function** | **95% CI** |
| --- | --- | --- | --- | --- | --- |
| Dorsiflexion peak torque (N·m) | 52 | −0.021 | −0.057, 0.016 | 0.198 | −0.155, 0.551 |
| Dorsiflexion peak torque/body mass (N·m·kg⁻¹) | 52 | −1.726 | −4.203, 0.752 | 0.209 | −0.091, 0.510 |
| Five-times sit-to-stand power/body mass (W·kg⁻¹) | 51 | −0.559 | −0.909, −0.209 | 0.484 | 0.181, 0.787 |
| Timed Up and Go (s) | 52 | 0.120 | 0.057, 0.182 | 0.442 | 0.212, 0.672 |
| Usual gait speed (m·s⁻¹) | 52 | −0.943 | −1.858, −0.027 | 0.292 | 0.008, 0.576 |
| Fast gait speed (m·s⁻¹) | 52 | −0.613 | −1.182, −0.044 | 0.311 | 0.022, 0.600 |
| Four-Square Step Test (s) | 51 | 0.054 | −0.027, 0.135 | 0.161 | −0.081, 0.403 |

**Notes.** The outcome was the participant-level change in force CoV, calculated as dual-task minus single-task CoV and expressed in percentage points. Original-unit slopes represent the expected CoV change per one-unit increase in the raw predictor. For torque, sit-to-stand power, and gait speed, lower raw values indicate poorer function; for Timed Up and Go and Four-Square Step Test, higher raw values indicate poorer function. Standardised slopes were recoded so that positive values consistently indicate a larger dual-task increase among participants with poorer function.

Table S5B. Robust coefficients of determination for the sex-adjusted change-score models.

| **Outcome** | **Full robust R²** | **Adjusted R²** | **Sex-only R²** | **Incremental R² beyond sex** |
| --- | --- | --- | --- | --- |
| Dorsiflexion peak torque (N·m) | 0.047 | 0.008 | 0.024 | 0.023 |
| Dorsiflexion peak torque/body mass (N·m·kg⁻¹) | 0.059 | 0.021 | 0.024 | 0.035 |
| Five-times sit-to-stand power/body mass (W·kg⁻¹) | 0.180 | 0.146 | 0.023 | 0.158 |
| Timed Up and Go (s) | 0.234 | 0.203 | 0.024 | 0.210 |
| Usual gait speed (m·s⁻¹) | 0.095 | 0.058 | 0.024 | 0.071 |
| Fast gait speed (m·s⁻¹) | 0.102 | 0.066 | 0.024 | 0.078 |
| Four-Square Step Test (s) | 0.049 | 0.009 | 0.017 | 0.032 |

**Notes.** The full model included the functional or strength predictor and sex. Incremental R² was calculated as the full-model robust R² minus the robust R² from the corresponding sex-only model. It therefore reflects the additional explanatory contribution of the predictor beyond sex.
