# Supplementary material for: Dual‐Tasking Exacerbates Force and Neural Control Unsteadiness in Older Adults With Sarcopenia
Source: J Cachexia Sarcopenia Muscle. 2026 Oct 5;17(5):e70397. doi: 10.1002/jcsm.70397 (PMC13638254; doi:10.1002/jcsm.70397)
Supplement: Supplementary file 4 [file JCSM-17-e70397-s004.docx]

Supplementary Material 7. Supplementary References

S41.	Vigotsky A, Halperin I, Lehman G, Trajano G, Vieira TM. Interpreting Surface Electromyography Studies in Sports and Rehabilitation Sciences. Front Physiol 2018;8:1–15.

S42.	Malmstrom TK, Miller DK, Simonsick EM, Ferrucci L, Morley JE. SARC-F: A symptom score to predict persons with sarcopenia at risk for poor functional outcomes. J Cachexia Sarcopenia Muscle 2016;7:28–36.

S43.	Holobar A, Zazula D. Multichannel blind source separation using convolution Kernel compensation. IEEE Transactions on Signal Processing 2007;55:4487–4496.

S44.	Frančič A, Holobar A. Motor Unit Tracking Across Low Contraction Levels of Biceps Brachii Muscle. In: Torricelli D, Metin A, Pons JL, editors. Converging Clinical and Engineering Research on Neurorehabilitation IV. Springer; 2022. pp. 401–405.

S45.	Negro F, Muceli S, Castronovo AM, Holobar A, Farina D. Multi-channel intramuscular and surface EMG decomposition by convolutive blind source separation. J Neural Eng 2016;13.

S46.	Gallet C, Julien C. The significance threshold for coherence when using the Welch’s periodogram method: Effect of overlapping segments. Biomed Signal Process Control 2011;6:405–409.

S47.	Baldissera F, Cavallari P, Cerri G. Motoneuronal pre-compensation for the low-pass filter characteristics of muscle. A quantitative appraisal in cat muscle units. Journal of Physiology 1998;611–627.

S48.	Beckwée D, Delaere A, Aelbrecht S, Baert V, Beaudart C, Bruyère O et al. Exercise interventions for the prevention and treatment of sarcopenia. A systematic umbrella review. J Nutr Health Aging 2019.

S49.	Khan MJ, Fong KNK, Wong TWL, Tsang WW nam, Chen C, Chan WC et al. Effectiveness of dual-task exercise in improving balance and preventing falls among older adults: systematic review with meta-analysis and meta-regression. Eur. Geriatr. Med. 2025;16:2047–2083.
