# Supplementary material for: Dual‐Tasking Exacerbates Force and Neural Control Unsteadiness in Older Adults With Sarcopenia
Source: J Cachexia Sarcopenia Muscle. 2026 Oct 5;17(5):e70397. doi: 10.1002/jcsm.70397 (PMC13638254; doi:10.1002/jcsm.70397)
Supplement: Supplementary file 5 [file JCSM-17-e70397-s005.docx]

Supplementary Material 3. Endurance-type vs Power-type athletes subgroup analysis.

**Torque and high-density electromyography outcomes**

*No differences between conditions and subgroups on torque coefficient of variation.* No subgroup [β = -0.27 (-0.77, 0.23), t = -1.05] or condition [β = 0.23 (-0.06, 0.05), t = 1.55], nor subgroup by condition interaction effects [β = 0.00 (-0.44, 0.44), t = -0.01] were observed for the torque coefficient of variation. No effect of sex was observed [β = -0.46 (-0.95, 0.02), t = -1.87]. Figure 3 displays torque coefficient of variation estimated marginal means and mean differences for each group and condition.

**Figure 3.** Effects of dual-tasking on torque coefficient of variation (CoV) across athletic subgroups. Panel A displays participant-level torque CoV during single-task and dual-task conditions for Endurance- and Power-type athletes. Each coloured point represents the mean discharge rate of one participant (blue = male, pink = female). Panel B displays condition contrasts (dual-task – single-task) shown separately for each subgroup. Torque CoV remained unchanged for both subgroups under dual-tasking. Panel C displays subgroup contrasts, shown separately for dual- and single-tasks conditions. No subgroup differences were observed. For all panels, black circles and error bars indicate estimated marginal means ± 95% confidence intervals.

*Mean discharge rates are increased in both endurance and power-type athletes during dual tasks.* A group by condition interaction was observed for mean discharge rates [β = -0.13 (-0.20, -0.07), t = 4.21]. Mean discharge rates increased for both subgroups, while no between subgroups and conditions differences were observed. Effects of recruitment threshold [β = -0.04 (-0.05, 0.04), t = -20.6] and sex were observed [β = -3.57 (-5.54, -1.60), t = -3.55]. Males had lower mean discharge rates than females. Figure 4 displays mean discharge rates estimated marginal means and mean differences for each subgroup and condition.

**Figure 4.** Effects of dual-tasking on mean motor unit discharge rates across subgroups. Panel A displays participant-level mean discharge rates (pps) during single-task and dual-task conditions for endurance- and power-type athletes. Each coloured point represents the mean discharge rate of one participant (blue = male, pink = female). Grey lines connect the same participant across conditions. Panel B displays condition contrasts (dual-task – single-task) shown separately for each subgroup. Mean discharge rates were significantly increased during dual-tasking compared with single-tasking across subgroups. Panel C displays subgroup contrasts, shown separately for dual- and single-task conditions. No significant subgroup differences were detected in either condition. For all panels, black circles and error bars indicate estimated marginal means ± 95% confidence intervals.

*Inter-spike intervals coefficient of variation decreased for both endurance- and power-type athletes and was higher for power-type athletes.* Subgroup [β = 2.47 (0.39, 4.54), t = 2.33] and condition effects were observed for inter-spike interval coefficient of variation [β = 0.65 (0.49, 0.80), t = 8.05]. Inter-spike interval coefficient of variation decreased for endurance- and power-type athletes. The power-type athletes displayed higher values than endurance in both single and dual tasks. An effect of recruitment threshold was observed [β = 0.10 (0.08, 0.11), t = 14.2], while no effect of sex was detected [β = -0.39 (-2.60, 1.82), t = -0.35]. Figure 5 displays inter-spike interval coefficient of variation estimated marginal means and mean differences for each subgroup and condition.

**Figure 5.** Effects of dual tasking on inter-spike interval variability across subgroups. Panel A displays participant-level inter-spike interval coefficient of variation (CoV, %) during single-task and dual-task conditions for endurance- and power-type subgroups. Each coloured point represents the mean value for one participant (blue = male, pink = female). Grey lines connect the same participant across conditions. Panel B displays condition contrasts (dual-task – single-task) shown separately for each subgroup. Inter-spike interval CoV was significantly reduced in both subgroups. Panel C displays subgroup contrasts. Endurance-type athletes displayed lower inter-spike interval coefficient of variation than power-type athletes. For all panels, black circles and error bars indicate estimated marginal means ± 95% confidence intervals.

*Delta-band coherence is reduced in power-type but not in endurance-type athletes during dual task.* A condition by subgroup interaction effect was observed for delta-band coherence [β = 6.76 (0.12, 13.4), t = 2.0]. Delta-band coherence reduced, for power-type athletes only, during dual task, with no subgroup differences at any condition. No effect of sex was observed [β = -10.8 (-26.4, 4.7), t = -1.37]. Figure 6 displays delta-band coherence estimated marginal means and mean differences for each subgroup and condition.

**Figure 6.** Effects of dual-tasking on delta-band coherence across subgroups. Panel A displays participant-level delta-band coherence (AUC) during single-task and dual-task conditions for endurance- and power-type subgroups. Each coloured point represents the mean value for one participant (blue = male, pink = female). Panel B displays condition contrasts (dual-task – single-task), collapsed across groups. Delta-band coherence was significantly reduced during dual-tasking compared with single-tasking for Power-type athletes only. Panel C displays group contrasts. No significant differences were observed between groups in either condition. For all panels, black circles and error bars indicate estimated marginal means ± 95% confidence intervals.

*Alpha-band coherence is higher in Power-type athlete but unchanged in both groups during dual task.* A subgroup effect was observed for alpha-band coherence [β = 24.0 (6.20, 41.8), t = 2.64]. Power-type displayed higher alpha-band coherence than endurance-type athletes. An effect of sex was observed, with males displaying lower alpha-band coherence than females [β = -19.9 (-38.5, -1.28), t = -2.09]. Figure 7 displays Alpha-band coherence estimated marginal means and mean differences for each group and condition.

Figure 7. Effects of dual-tasking on alpha-band coherence across subgroups. Panel A displays participant-level alpha-band coherence (AUC) during single-task and dual-task conditions. Each coloured point represents the mean value for one participant (blue = male, pink = female). Panel B displays condition contrasts (dual-task – single-task) shown separately for each group. Alpha-band coherence remained unchanged for both groups. Panel C displays group contrast. Alpha-band coherence was higher for Power- than Endurance-type athletes. For all panels, black circles and error bars indicate estimated marginal means ± 95% confidence intervals.

*Beta-band coherence is reduced for endurance-type athletes during dual tasks.* A subgroup by condition interaction was observed for Beta-band coherence [β = -8.9 (-17.4, -0.3), t = -2.03]. Beta-band coherence reduced during the dual task for the endurance-type subgroup only, and was higher for endurance- than power-type at the dual-task condition. No effect of sex was observed [β = -4.76 (-29.5, 20.0), t = -0.38]. Figure 8 displays Beta-band coherence estimated marginal means and mean differences for each group and condition.

**Figure 8.** Effects of dual-tasking on beta-band coherence across subgroups. Panel A displays participant-level beta-band coherence (AUC) during single-task and dual-task conditions for endurance- and power-type athletes. Each coloured point represents the mean value for one participant (blue = male, pink = female). Panel B displays condition contrasts (dual-task – single-task) shown separately for each group. Beta-band coherence was significantly reduced for endurance-type athletes during dual task. Panel C displays group contrasts, shown separately for dual- and single-task conditions. Endurance-type athletes displayed higher beta-band coherence than power-type during the dual-task condition. For all panels, black circles and error bars indicate estimated marginal means ± 95% confidence intervals.
