# Supplementary material for: Dual‐Tasking Exacerbates Force and Neural Control Unsteadiness in Older Adults With Sarcopenia
Source: J Cachexia Sarcopenia Muscle. 2026 Oct 5;17(5):e70397. doi: 10.1002/jcsm.70397 (PMC13638254; doi:10.1002/jcsm.70397)
Supplement: Supplementary file 6 [file JCSM-17-e70397-s006.docx]

Supplementary Material 1. Plain-language guide to neuromuscular outcomes.

This table summarises how each study outcome was derived, its physiological interpretation, and its potential functional relevance.

| Outcome | How it was measured or calculated | What it is thought to represent physiologically | Potential functional relevance | Interpretation |
| --- | --- | --- | --- | --- |
| Torque coefficient of variation (torque CoV) | The standard deviation of torque divided by mean torque during the approximately 18-s analysis window, multiplied by 100. | Variability of the mechanical torque output produced by the participant. | Steady torque production is important for precise movement, postural control, and controlled interaction with the environment. Greater variability may indicate reduced ability to maintain stable motor output. | Higher values indicate poorer force steadiness. |
| Mean motor-unit discharge rate | Instantaneous discharge rate was calculated as the inverse of each inter-spike interval. Rates were then averaged across the analysis window for each motor unit. | The average firing frequency of the sampled motor units. It reflects one component of motor-unit rate coding. | Changes in discharge rate contribute to the regulation and maintenance of muscle force. In this study, it also helped determine whether changes in steadiness or coherence occurred alongside changes in average motor-unit firing rate. | Higher values indicate more frequent discharge by the sampled motor units, but are not inherently better or worse. |
| Inter-spike interval coefficient of variation (inter-spike interval CoV) | The standard deviation of the intervals between consecutive motor-unit discharges divided by their mean, multiplied by 100. | The temporal regularity of individual motor-unit discharge. It does not distinguish common from independent sources of variability or provide their frequency content. | More regular motor-unit discharge generally supports stable force production. Greater discharge variability may contribute to larger force fluctuations, particularly during low-force tasks. | Higher values indicate less regular motor-unit discharge and poorer neural control steadiness. |
| Intramuscular motor-unit coherence | Motor-unit discharge trains were summed into two cumulative spike trains, each containing five randomly selected motor units. Coherence between the two cumulative spike trains was calculated in the frequency domain, repeated across up to 100 permutations, transformed to z-scores, and expressed as area under the curve within predefined frequency bands. | The strength of frequency-specific oscillatory input shared across multiple motor units. It describes how common synaptic input to the motoneuron pool is organised. Coherence does not identify a specific anatomical source. | Shared input coordinates motor-unit activity and may influence the smoothness, consistency, and adaptability of force production. Different frequency bands capture different temporal components of this shared input. | Higher coherence indicates stronger shared oscillatory activity within that frequency band. Higher or lower values are not inherently beneficial. |
| Delta-band coherence (1-5 hz) | Area under the z-transformed coherence curve between 1 and 5 Hz. | Low-frequency common input commonly associated with slow force fluctuations and the effective common drive transmitted to the motoneuron pool. | Delta-band input contributes to low-frequency modulation of motor-unit activity during sustained force. Changes do not necessarily correspond directly to better or worse steadiness because force reflects multiple common and independent inputs. | Greater values indicate stronger low-frequency common input. Interpretation should consider torque variability and the other coherence bands. |
| Alpha-band coherence (5-15 hz) | Area under the z-transformed coherence curve between 5 and 15 Hz. | Frequency-specific common input that may include physiological tremor-related, spinal, and afferent contributions. | Oscillatory input in this range may be transmitted to the force signal and contribute to unwanted force fluctuations or physiological tremor. An increase may therefore be relevant to reduced precision of force control. | Greater values indicate stronger shared oscillatory input in the alpha range. In this study, increases coincided with poorer discharge and force steadiness in the sarcopenic group. |
| Beta-band coherence (15-35 hz) | Area under the z-transformed coherence curve between 15 and 35 Hz. | Frequency-specific common input commonly associated with corticospinal contributions, although it cannot definitively localise the input to the motor cortex or corticospinal pathway. | Beta-band activity may contribute to coordination of motor-unit activity and adaptation of descending motor commands during sustained force production. Its functional meaning depends on the task and broader pattern of neural activity. | Greater values indicate stronger shared beta-band oscillations. |
| Motor-unit recruitment threshold | Torque, expressed as a percentage of maximal voluntary torque, at the first identified discharge of each motor unit. | The force level at which a motor unit becomes active. It partly reflects the position of the sampled unit within the recruitment hierarchy. | Earlier- and later-recruited motor units can have different discharge characteristics. Recruitment threshold was therefore included as a covariate in analyses of motor-unit outcomes. | Lower values indicate earlier recruitment; higher values indicate recruitment at a greater relative force. It was not a primary functional outcome. |
