# Supplementary material for: Dual‐Tasking Exacerbates Force and Neural Control Unsteadiness in Older Adults With Sarcopenia
Source: J Cachexia Sarcopenia Muscle. 2026 Oct 5;17(5):e70397. doi: 10.1002/jcsm.70397 (PMC13638254; doi:10.1002/jcsm.70397)
Supplement: Supplementary file 7 [file JCSM-17-e70397-s007.docx]

Supplementary Material 2. Sensitivity analyses of potential age effects

**Summary**

The groups differed in chronological age, but age-group overlap produced low variance inflation and did not destabilise the primary models. When phenotype group was omitted, older age was associated with modestly greater torque and inter-spike interval variability, but not with mean discharge rate or delta-, alpha-, or beta-band coherence. Importantly, adding age to the full group-by-condition models did not identify an age effect for any outcome and did not change the direction or interpretation of the principal group and dual-task effects. In an exploratory age-matched comparison, the key sarcopenic findings were retained: sarcopenic participants showed greater inter-spike interval variability in both conditions, greater torque variability during dual-tasking, sarcopenic-specific worsening of both steadiness outcomes, and sarcopenic-specific increases in alpha- and beta-band coherence during dual-tasking. Some contrasts in controls and for delta-band coherence no longer excluded zero in the smaller matched sample, reflecting reduced precision. The analyses indicate that chronological age alone does not adequately explain the phenotype-specific dual-task responses reported in the manuscript, although residual age confounding cannot be completely excluded because the groups were not prospectively age-matched.

**Statistical approach**

These secondary analyses used the same data preprocessing, robust linear mixed-effects framework, covariates, and random-effects structures as the primary analyses. Age-group overlap was quantified using the proportion of age variance explained by group (R², equivalent to eta-squared for this model), the correlation ratio (eta), and variance inflation factors.

**Age-group overlap and collinearity**

Group explained 24.4% of the variance in age [R² = 0.244; F(2,49) = 7.92, p = 0.001], corresponding to a correlation ratio of eta = 0.494. Pairwise estimates showed a clear age difference between masters athletes and sarcopenic participants, whereas the confidence intervals for athlete-control and control-sarcopenic differences included zero (Table S1). Despite the overall association between age and group, the variance inflation factors (VIF) for age induced by group was only 1.32. GVIFs were also low in the fixed-effects designs used for the force/coherence and motor-unit models (adjusted GVIF values 1.00 to 1.16; Table S2), providing no evidence of problematic multicollinearity.

**Table S1.** Age-group overlap metrics and pairwise mean age differences.

| **Component** | **Metric or contrast** | **Estimate** | **95% CI / test** |
| --- | --- | --- | --- |
| Overall overlap | Age variance explained by group (R² / eta²) | 0.244 | F(2,49) = 7.92; p = 0.001 |
| Overall overlap | Correlation ratio (eta) | 0.494 | - |
| Overall overlap | VIF for age induced by group | 1.32 | - |
| Pairwise age difference | Masters athletes - Controls | -4.51 years | -9.31 to 0.29 |
| Pairwise age difference | Masters athletes - Sarcopenia | -9.46 years | -15.27 to -3.66 |
| Pairwise age difference | Controls - Sarcopenia | -4.95 years | -10.62 to 0.71 |

**Table S2.** Generalised variance inflation factors for the fixed-effects designs. Values in parentheses are GVIF^(1/[2 × df]), which are interpreted on the conventional VIF scale.

| **Term** | **df** | **Force/coherence design: GVIF (adjusted)** | **Motor-unit design: GVIF (adjusted)** |
| --- | --- | --- | --- |
| Age | 1 | 1.338 (1.157) | 1.350 (1.162) |
| Group | 2 | 1.393 (1.086) | 1.458 (1.099) |
| Condition | 1 | 1.000 (1.000) | 1.000 (1.000) |
| Sex | 1 | 1.107 (1.052) | 1.125 (1.061) |
| Recruitment threshold | 1 | Not included | 1.030 (1.015) |

**Overall associations between age and outcomes in models excluding group**

To characterise the overall age-outcome relationship without conditioning on phenotype, each outcome was modelled with age and task condition while group was omitted. Recruitment threshold was retained for motor-unit outcomes. Older age was associated with greater inter-spike interval coefficient of variation [β = 0.159 percentage points per year, 95% CI 0.052 to 0.265, t = 2.92] and greater torque coefficient of variation [β = 0.051 percentage points per year, 95% CI 0.010 to 0.092, t = 2.46]. Age was not associated with mean discharge rate or any coherence band (Table S3). These associations show that general age-related deterioration contributes to steadiness across the cohort, but they do not test whether age explains the group-specific dual-task responses. That question is addressed by the age-adjusted and age-matched analyses below.

**Table S3.** Age coefficients from robust mixed-effects models excluding phenotype group.

| **Outcome** | **Age coefficient per year** | **SE** | **t** | **95% CI** |
| --- | --- | --- | --- | --- |
| Mean discharge rate (pps) | -0.0351 | 0.0372 | -0.95 | -0.1079 to 0.0377 |
| Inter-spike interval CoV (percentage points) | 0.1589 | 0.0543 | 2.92 | 0.0524 to 0.2653 |
| Torque CoV (percentage points) | 0.0509 | 0.0207 | 2.46 | 0.0103 to 0.0915 |
| Delta-band coherence (AUC) | -0.1289 | 0.3905 | -0.33 | -0.8942 to 0.6364 |
| Alpha-band coherence (AUC) | -0.5369 | 0.5241 | -1.02 | -1.5641 to 0.4903 |
| Beta-band coherence (AUC) | -0.3180 | 0.4149 | -0.77 | -1.1312 to 0.4953 |

**Exploratory age-matched sarcopenia-control analysis**

Each of the 11 sarcopenic participants was matched 1:1 to a control using greedy nearest-neighbour matching on age without replacement, with the oldest sarcopenic participants matched first. The mean absolute within-pair age difference was 1.64 years. Ten of the 11 pairs differed by no more than 2 years; the 97-year-old sarcopenic participant was matched to the oldest available control aged 86 years. After matching, mean age was 78.3 ± 5.5 years in controls and 79.7 ± 7.8 years in sarcopenic, and the between-group age difference did not exclude zero [Control - Sarcopenic = -1.46 years, 95% CI -7.48 to 4.57]. The matched analysis is therefore exploratory and should be interpreted as a robustness check rather than a definitive age-controlled comparison.

**Table S4.** Age balance in the exploratory matched sample.

| **Group** | **n** | **Mean age ± SD (years)** | **Age range** |
| --- | --- | --- | --- |
| Controls | 11 | 78.3 ± 5.5 | 69-86 |
| Sarcopenia | 11 | 79.7 ± 7.8 | 69-97 |

The matched models retained the main-model fixed effects (sex and, for motor-unit outcomes, recruitment threshold) and random-effects structure. Group contrasts are Control - Sarcopenic; therefore, negative estimates indicate higher values in sarcopenic individuals. Condition contrasts are Dual-task - Single-task; positive estimates indicate an increase during dual-tasking.

**Table S5.** Control-sarcopenia contrasts within each condition in the age-matched sample.

| **Outcome** | **Condition** | **Control - Sarcopenia** | **95% CI** |
| --- | --- | --- | --- |
| Mean discharge rate (pps) | Dual-task | 0.7853 | -0.5257 to 2.0963 |
| Mean discharge rate (pps) | Single-task | 0.6208 | -0.6903 to 1.9319 |
| Inter-spike interval CoV (percentage points) | Dual-task | -3.6862 | -6.0313 to -1.3411 |
| Inter-spike interval CoV (percentage points) | Single-task | -2.6697 | -5.0156 to -0.3238 |
| Torque CoV (percentage points) | Dual-task | -2.0014 | -3.3779 to -0.6248 |
| Torque CoV (percentage points) | Single-task | -0.9200 | -2.2948 to 0.4549 |
| Delta-band coherence (AUC) | Dual-task | 0.3489 | -16.8487 to 17.5465 |
| Delta-band coherence (AUC) | Single-task | 2.3928 | -14.7961 to 19.5818 |
| Alpha-band coherence (AUC) | Dual-task | -5.5289 | -24.3515 to 13.2938 |
| Alpha-band coherence (AUC) | Single-task | 9.8137 | -8.9963 to 28.6238 |
| Beta-band coherence (AUC) | Dual-task | -15.7702 | -27.8957 to -3.6447 |
| Beta-band coherence (AUC) | Single-task | -8.5702 | -20.6779 to 3.5375 |

**Table S6.** Dual-task - single-task contrasts within each group in the age-matched sample.

| **Outcome** | **Group** | **Dual-task - Single-task** | **95% CI** |
| --- | --- | --- | --- |
| Mean discharge rate (pps) | Controls | 0.3213 | 0.2685 to 0.3740 |
| Mean discharge rate (pps) | Sarcopenia | 0.1568 | 0.0990 to 0.2145 |
| Inter-spike interval CoV (percentage points) | Controls | -0.0885 | -0.3152 to 0.1381 |
| Inter-spike interval CoV (percentage points) | Sarcopenia | 0.9280 | 0.6793 to 1.1767 |
| Torque CoV (percentage points) | Controls | 0.1282 | -0.4003 to 0.6568 |
| Torque CoV (percentage points) | Sarcopenia | 1.2096 | 0.6845 to 1.7347 |
| Delta-band coherence (AUC) | Controls | -4.1853 | -8.4160 to 0.0455 |
| Delta-band coherence (AUC) | Sarcopenia | -2.1414 | -6.3449 to 2.0622 |
| Alpha-band coherence (AUC) | Controls | -5.3133 | -10.6636 to 0.0371 |
| Alpha-band coherence (AUC) | Sarcopenia | 10.0294 | 4.7136 to 15.3451 |
| Beta-band coherence (AUC) | Controls | -1.0011 | -6.1131 to 4.1109 |
| Beta-band coherence (AUC) | Sarcopenia | 6.1989 | 1.1204 to 11.2774 |

**Interpretation of the matched analysis**

Age matching reproduced the central sarcopenic-specific findings. Mean discharge rate increased during dual-tasking in both groups and did not differ between groups. Sarcopenic participants retained higher inter-spike interval variability in both conditions, and only sarcopenic showed increased inter-spike interval and torque variability during dual-tasking. The between-group torque difference remained evident during dual-tasking but not during the single-task condition. Sarcopenic-specific increases in alpha- and beta-band coherence also remained, with beta-band coherence higher in sarcopenic than controls during dual-tasking. Delta-band condition effects and the decreases in alpha- or beta-band coherence among controls did not exclude zero in this smaller sample. Thus, the matched analysis supports the interpretation that the deterioration in steadiness and the divergent alpha- and beta-band responses under cognitive load are not attributable solely to the older mean age of the sarcopenic group, while also showing the loss of precision expected after reducing the control sample from 22 to 11 participants.

**Primary models additionally adjusted for age**

**Torque and high-density electromyography outcomes**

*Torque steadiness was reduced in sarcopenia and worsens during dual tasking.* Force control was less steady (i.e., higher torque coefficient of variation) in the sarcopenic group than in controls and athletes during both single- and dual-task conditions (Figure s1). Force steadiness worsened during the dual-task condition in the sarcopenic group only, whereas no condition differences were observed in controls or athletes. Figure s1 shows estimated marginal means and mean differences in torque coefficient of variation (a proxy of force steadiness) for each group and condition. The statistical model identified a group by condition interaction [β = -1.37 (-1.84, -0.91), t = -5.76], with no effect of age [β = 0.01 (-0.03, 0.05), t = 0.43].

**Figure s1.** Effects of dual-tasking on torque coefficient of variation (CoV) across groups. Panel A displays participant-level torque CoV during single-task and dual-task conditions for Athlete, Control, and Sarcopenia groups. Each coloured point represents the mean discharge rate of one participant (blue = male, pink = female). Grey lines connect the same participant across conditions. Panel B displays condition contrasts (dual-task – single-task) shown separately for each group. Torque CoV remained unchanged for athletes and controls, and increased markedly for sarcopenic individuals under dual-tasking. Panel C displays group contrasts, shown separately for dual- and single-tasks conditions. Under dual-tasking, sarcopenic individuals exhibited significantly higher torque CoV compared with both athletes and controls. For all panels, black circles and error bars indicate estimated marginal means ± 95% confidence intervals.

*Mean discharge rates increased in all groups during dual tasking.* Mean discharge rates, a proxy of the neural drive to the muscle, increased during the dual-task condition in all groups, with no differences observed between sarcopenic, control, and athlete participants in either condition. Figure s2 shows estimated marginal means and mean differences in mean discharge rate for each group and condition. The statistical model identified a group by condition interaction [β = 0.27 (0.20, 0.33), t = 8.16]. Effects of recruitment threshold [β = -0.031 (-0.034, -0.028), t = -20.0] and sex [β = -1.3 (-2.4, -0.3), t = -2.51] were observed, indicating that motor units recruited at higher thresholds discharged at lower rates, and that males displayed lower mean discharge rates than females. No effect of age was observed [β = -0.04 (-0.12, 0.05), t = -0.87].

**Figure s2.** Effects of dual-tasking on mean motor unit discharge rates across groups. Panel A displays participant-level mean discharge rates (pps) during single-task and dual-task conditions for Athlete, Control, and Sarcopenia groups. Each coloured point represents the mean discharge rate of one participant (blue = male, pink = female). Grey lines connect the same participant across conditions. Panel B displays condition contrasts (dual-task – single-task) shown separately for each group. Mean discharge rates were significantly increased during dual-tasking compared with single-tasking across all groups. Panel C displays group contrasts, shown separately for dual- and single-task conditions. No significant group differences were detected in either condition. For all panels, black circles and error bars indicate estimated marginal means ± 95% confidence intervals.

*Neural control steadiness worsened in sarcopenic individuals, improved in athletes, and remained unchanged in controls during dual-tasking.* Neural control was less steady (i.e., higher inter-spike interval coefficient of variation) in the sarcopenic group than in controls and athletes during both single- and dual-task conditions (Figure 5). Neural drive steadiness worsened during the dual-task condition in the sarcopenic group, improved in athletes, and remained unchanged in controls. Athletes displayed more stable neural drive (lower coefficient of variation) than controls during the dual-task condition only. Figure 5 shows estimated marginal means and mean differences in inter-spike interval coefficient of variation (as a proxy of neural drive steadiness) for each group and condition. The statistical model identified a group by condition interaction [β = -1.53 (-1.80, -1.27), t = -11.3]. An effect of recruitment threshold was observed [β = 0.14 (0.13, 0.15), t = 27], while no effect of age was detected [β = 0.05 (-0.08, 0.18), t = 0.76].

**Figure 5.** Effects of dual-tasking on inter-spike interval variability across groups. Panel A displays participant-level inter-spike interval coefficient of variation (CoV, %) during single-task and dual-task conditions for Athlete, Control, and Sarcopenia groups. Each coloured point represents the mean value for one participant (blue = male, pink = female). Grey lines connect the same participant across conditions. Panel B displays condition contrasts (dual-task – single-task) shown separately for each group. Inter-spike interval CoV was significantly reduced in athletes, remained unchanged in controls, and increased in sarcopenic individuals under dual-tasking. Panel C displays group contrasts, shown separately for dual- and single-task conditions. Under dual-tasking, sarcopenic individuals exhibited significantly higher inter-spike interval CoV compared with both athletes and controls, whereas athletes demonstrated lower variability than controls. For all panels, black circles and error bars indicate estimated marginal means ± 95% confidence intervals.

*Common input associated with force fluctuations was reduced in all groups during dual-tasking.* Common neural input related to force fluctuations (delta-band) was reduced during the dual-task condition in all groups, with no differences observed between sarcopenic, control, and athlete participants in either condition. Figure 6 shows estimated marginal means and mean differences in delta-band coherence for each group and condition. The statistical model identified a condition effect [β = 5.55 (2.32, 8.77), t = 3.37], with no effect of age [β = -0.43 (-1.27, 0.40), t = -1.01].

**Figure 6.** Effects of dual-tasking on delta-band coherence across groups. Panel A displays participant-level delta-band coherence area under the curve (AUC) during single-task and dual-task conditions for Athlete, Control, and Sarcopenia groups. Each coloured point represents the mean value for one participant (blue = male, pink = female). Panel B displays condition contrasts (dual-task – single-task), collapsed across groups. Delta-band coherence was significantly reduced during dual-tasking compared with single-tasking across all groups. Panel C displays group contrasts, collapsed across conditions. No significant differences were observed between groups in either condition. For all panels, black circles and error bars indicate estimated marginal means ± 95% confidence intervals.

*Involuntary physiological tremor fluctuations in common input increased in the sarcopenic group during dual-tasking.* Neural input associated to involuntary physiological tremor contributions to common input (alpha-band coherence) increased during the dual-task condition in the sarcopenic group only (Figure 7). No changes were observed between conditions in controls or athletes, and no differences between these groups were detected in either condition. Figure 7 shows estimated marginal means and mean differences in alpha-band coherence for each group and condition. The statistical model identified a group by condition interaction [β = -12.4 (-18.8, -6.08), t = -3.84], with no effect of age [β = -0.64 (-1.84, 0.55), t = -1.05].

**Figure 7.** Effects of dual-tasking on alpha-band coherence across groups. Panel A displays participant-level alpha-band coherence area under the curve (AUC) during single-task and dual-task conditions for Athlete, Control, and Sarcopenia groups. Each coloured point represents the mean value for one participant (blue = male, pink = female). Panel B displays condition contrasts (dual-task – single-task) shown separately for each group. Alpha-band coherence remained unchanged in athletes and controls, but increased in sarcopenic individuals during dual-tasking. Panel C displays group contrasts, shown separately for dual- and single-task conditions. No significant differences were observed between groups in either condition. For all panels, black circles and error bars indicate estimated marginal means ± 95% confidence intervals.

*Corticospinal coupling was increased in the sarcopenic group, and reduced in controls and athletes during dual-tasking.* Neural input reflecting corticospinal contributions to common drive (beta-band coherence) increased during the dual-task condition in the sarcopenic group, but decreased in controls and athletes. Figure 8 shows estimated marginal means and mean differences in beta-band coherence for each group and condition. The statistical model identified a group by condition interaction [β = -13.3 (-20.0, -6.60), t = -3.89], with no effect of age [β = -0.47 (-1.42, 0.49), t = -0.96].

**Figure 8.** Effects of dual-tasking on beta-band coherence across groups. Panel A displays participant-level beta-band coherence area under the curve (AUC) during single-task and dual-task conditions for Athlete, Control, and Sarcopenia groups. Each coloured point represents the mean value for one participant (blue = male, pink = female). Panel B displays condition contrasts (dual-task – single-task) shown separately for each group. Beta-band coherence was significantly reduced in athletes and controls under dual-tasking, but increased in sarcopenic individuals. Panel C displays group contrasts, shown separately for dual- and single-task conditions. No significant differences were observed between groups in either condition. For all panels, black circles and error bars indicate estimated marginal means ± 95% confidence intervals.
